# The prion-like protein kinase Sky1 is required for efficient stress granule disassembly

**DOI:** 10.1038/s41467-019-11550-w

**Published:** 2019-08-09

**Authors:** Jenifer E. Shattuck, Kacy R. Paul, Sean M. Cascarina, Eric D. Ross

**Affiliations:** 1 Department of Biochemistry and Molecular Biology, Colorado State University, Fort Collins, CO 80523 USA; 2 Present Address: Department of Pharmacology and Experimental Therapeutics, Boston University School of Medicine, Boston, MA 02118 USA

**Keywords:** Kinases, Prions, Protein aggregation, Stress granules

## Abstract

Stress granules are membraneless protein- and mRNA-rich organelles that form in response to perturbations in environmental conditions. Stress granule formation is reversible, and persistent stress granules have been implicated in a variety of neurodegenerative disorders, including amyotrophic lateral sclerosis. However, characterization of the factors involved in dissolving stress granules is incomplete. Many stress granule proteins contain prion-like domains (PrLDs), some of which have been linked to stress granule formation. Here, we demonstrate that the PrLD-containing yeast protein kinase Sky1 is a stress granule component. Sky1 is recruited to stress granules in part via its PrLD, and Sky1’s kinase activity regulates timely stress granule disassembly during stress recovery. This effect is mediated by phosphorylation of the stress granule component Npl3. Sky1 can compensate for defects in chaperone-mediated stress granule disassembly and vice-versa, demonstrating that cells have multiple overlapping mechanisms for re-solubilizing stress granule components.

## Abstract (web summary)

The factors regulating stress granule dissolution are not fully understood. Here, the authors identify Sky1 as a stress granule component in yeast, and show that Sky1 kinase activity is required for timely stress granule disassembly during stress recovery.

## Introduction

Stress granules form in the cytoplasm when translation initiation is limiting, including under a variety of stress conditions^1^. Stress granules are composed of mRNAs and protein, and contain mRNA-protein complexes (mRNPs) stalled in translation initiation. These cytoplasmic granules are important for cellular homeostasis, and mutations that increase stress granule formation or decrease clearance have been linked to various neurodegenerative diseases, as well as some cancers^2,3^. Stress granules are part of a growing class of nonmembranous organelles that includes processing bodies, nuclear speckles, and nucleoli^4^. While all of these assemblies are dynamic protein–RNA complexes, they differ in their composition and function.

Stress granules show liquid-like properties, and are thought to form at least in part by liquid–liquid phase separation (LLPS)^1^. Many of the RNA-binding proteins found in stress granules contain intrinsically disordered low-complexity domains; some of these low-complexity domains are prion-like domains (PrLDs), defined as protein domains that compositionally resemble yeast prion domains^5^. In vitro, many of these PrLDs can undergo LLPS. As untranslated mRNPs assemble into stress granules, this concentrates low-complexity domains; it is hypothesized that these low-complexity domains create a liquid phase, thereby recruiting other low-complexity domains by LLPS^6^.

While it is known that stress granules are highly dynamic and reversible, the mechanisms of regulation of granule resolution are not fully understood. Stress granules can be degraded by autophagy^7^. In addition, various mechanisms for dissolution have been proposed. A variety of evidence indicates that protein quality control systems play a role in disassembly^8–11^. RNA/DNA helicases have been proposed to promote disassembly by utilizing ATP hydrolysis to disrupt RNA–protein interactions^12,13^. Posttranslational modifications including methylation and phosphorylation also influence stress granule assembly by altering protein–protein interactions (reviewed in^1,14,15^), so similar modifications may likewise influence granule dissolution. These mechanisms are not mutually exclusive, and since different stresses result in granules with different protein compositions^16,17^, the mechanisms of dissolution may vary depending on the type of stress.

Here we provide evidence that the PrLD-containing yeast protein kinase Sky1 acts as a regulator of stress granule dissolution. We find that Sky1 localizes to heat-induced stress granules, and that its PrLD promotes this recruitment. Sky1’s kinase activity is necessary for efficient disassembly of stress granules. Sky1 promotes granule dissolution at least in part through phosphorylation of Npl3, a nucleocytoplasmic mRNA shuttling protein. Finally, when Sky1 is overexpressed, it partially compensates for defects in other disassembly pathways, highlighting the extent to which overlapping pathways contribute to stress granule disassembly.

## Results

### Sky1 is a stress granule component

**Fig. 1:**
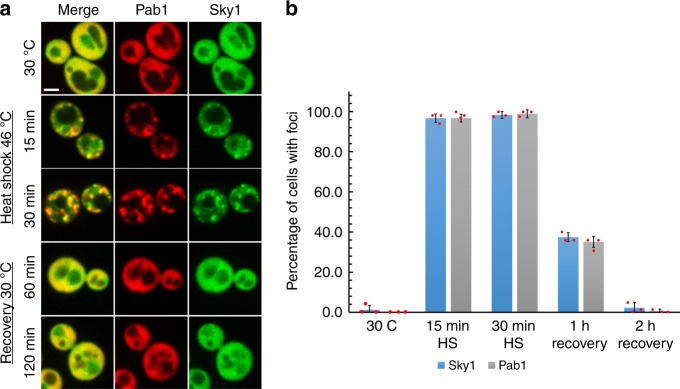
Sky1 is a stress granule protein. **a** Sky1 colocalizes with Pab1 foci during heat stress. Yeast expressing Sky1-GFP and Pab1-mCherry from the corresponding endogenous loci were grown at 30 °C to mid-log, subjected to heat shock at 46 °C, and allowed to recover at 30 °C. Cells were visualized by fluorescence microscopy during heat shock and recovery. Scale bar, 2 μm. **b** Quantification of the percentage of cells with Pab1 (gray bar) or Sky1 (blue bar) foci. Data represent means ± SD of *n* = 3 independent replicates. Source data are provided as a Source Data file

A recent mass spectrometry study characterized protein components of heat-induced protein aggregates, which included stress granules, in the yeast *Saccharomyces cerevisiae*^18^. We noticed that multiple known or proposed targets of the protein kinase Sky1 were found in heat-induced granules^19–21^. We therefore hypothesized that Sky1 might act as a regulator of stress granules. None of the previous studies characterizing protein components of yeast stress granules identified Sky1 as a component^18,22–25^. However, these lists are unlikely to be comprehensive. Some components may be lost during the purification process, poorly detected, or unique to specific stress conditions. To test whether Sky1 is recruited into heat-induced stress granules, we subjected *S. cerevisiae* cells to heat shock at 46 °C for 15 or 30 min, and tested for colocalization between Sky1 and known stress granule proteins. After either 15 or 30 min of heat shock, Sky1-GFP was found in foci (Fig. 1). These foci largely showed a high degree of colocalization (85 ± 12% after 30 min of heat shock; data represent means ± SD of *n* = 3 independent replicates) with foci formed by mCherry-tagged Pab1, a marker of stress granules^26^. Colocalization was also seen with cherry-tagged versions of the stress granule proteins Tif4631, Pub1, and Ded1 (Supplementary Fig. 1a).

After 30 min of heat shock, cells were allowed to recover at 30 °C. After 1 h recovery, ~60% of the cells showed diffuse Sky1-GFP signal, and almost all cells showed diffuse signal after 2 h. Each of the stress granule markers showed slightly different rates of resolubilization during recovery, but each showed almost entirely diffuse signal by 2 h (Fig. 1b, Supplementary Fig. 1b).

Stress granule formation is inhibited by cycloheximide, a chemical that stalls ribosomes by blocking translation elongation^27^. Addition of cycloheximide substantially reduced foci formation by Sky1-GFP and Pab1-mCherry (Supplementary Fig. 1c). Collectively, these data indicate that Sky1 is indeed a component of stress granules.

### Sky1’s PrLD promotes recruitment to stress granules

**Fig. 2:**
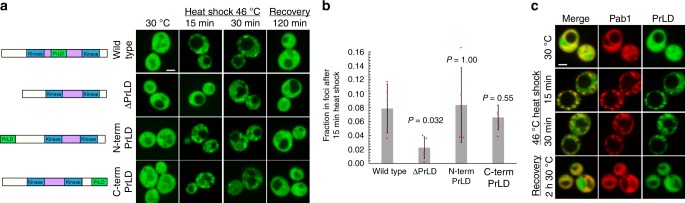
Sky1’s PrLD promotes Sky1 recruitment to stress granules. **a** Analysis of yeast expressing from the endogenous *SKY1* locus GFP-tagged Sky1 mutants in which the core PrLD (amino acids 388–457) was deleted (∆PrLD), or moved to the N terminus (N term PrLD) or C terminus (C term PrLD). Cells were grown to mid-log phase and subjected to heat shock and recovery. A schematic of each mutant is shown. Scale bar, 2 μm. **b** Quantification of the fraction of Sky1 in stress granule foci after 15 min of heat shock. Data represent mean ± SD from of *n* = 5 independent replicates. A two-sided Mann–Whitney *U* test was used to calculate *P* values. **c** The Sky1 PrLD is sufficient to associate with stress granules. Yeast expressing Pab1-mCherry from the endogenous locus were transformed with plasmids expressing the Sky1 PrLD fused to GFP, under control of the SUP35 promoter. Cells were visualized by fluorescence microscopy during heat shock at 46 °C and recovery at 30 °C. Scale bar, 2 μm. Source data are provided as a Source Data file

We previously showed that Sky1 contains an aggregation-prone PrLD^28^. When overexpressed, the PrLD forms SDS-insoluble cytoplasmic aggregates^28^. The PrLD of the human protein TIA-1 is required for stress granule formation, and the prion domain of the yeast prion protein Sup35 can substitute for the TIA-1 PrLD in supporting stress granule formation^29^. We therefore tested if the PrLD was similarly necessary for Sky1 recruitment into stress granules. Deletion of the core PrLD did not entirely eliminate foci formation by Sky1-GFP, but did result in a statistically significant reduction in the fraction of Sky1 in foci upon heat stress (Fig. 2a, b).

One possible explanation for the reduction in granule recruitment is that PrLD deletion could perturb the normal structure of Sky1. This seemed unlikely; the PrLD falls within a spacer region between the two kinase domains of Sky1, and Sky1 is still functional and normally folded when the entire spacer domain is deleted^30^. Consistent with normal folding, a Sky^∆PrLD^ mutant showed similar protein levels to wild-type Sky1 (Supplementary Fig. 2a). To further confirm that the deletion did not compromise Sky1 activity, we monitored the localization of Npl3, a known phosphorylation target of Sky1. Npl3 is a mRNA shuttling protein that is phosphorylated by Sky1 in the cytoplasm to release its mRNA and return Npl3 to the nucleus^31^. Under steady state conditions, Npl3 is predominantly in the nucleus (Supplementary Fig. 2b). A K187M mutation in the Sky1 ATP binding site inactivates Sky1’s kinase activity^32^; replacement of the *SKY1* open reading frame with a GFP-tagged *sky1*^*K187M*^ allele resulted in increased cytoplasmic localization of Npl3 (Supplementary Fig. 2b), consistent with previous reports^31^. By contrast, the deletion of the Sky1 PrLD did not alter Npl3’s nuclear localization (Supplementary Fig. 2b). These results indicate that PrLD deletion reduces Sky1 stress granule recruitment, but does not substantially affect Sky1’s kinase activity.

While our experiments link the PrLD to stress granule recruitment, they do not prove that the assembly activity of the PrLD is responsible for recruitment. PrLD deletions could have structural or steric effects on the protein that are not detected in activity assays. Likewise, in replacement experiments (analogous to those replacing the TIA-1 PrLD with the Sup35 prion domain), the replacement could restore recruitment because it satisfies these structural or steric needs, rather than because of its assembly activity. If these activities are truly driven by the assembly activity of the PrLD, we would make two additional predictions. First, the aggregation activity of PrLDs is generally modular, meaning that PrLDs maintain aggregation activity when transferred to other proteins^33^. Therefore, we would predict similar modular behavior for stress granule recruitment. Second, while assembly-prone PrLDs should be able to replace the PrLD in supporting granule recruitment, PrLDs designed to have low assembly activity should not. Therefore, to more directly link PrLD assembly to granule recruitment, we tested these predictions.

To determine whether the Sky1 PrLD acts in a modular manner to promote stress granule recruitment, we tested whether this activity was independent of the position of the PrLD within Sky1. Indeed, moving the PrLD to the N terminus or C terminus did not significantly affect Sky1’s assembly (Fig. 2b) or localization to granules (Supplementary Fig. 2c) in response to heat. These mutations also did not affect Npl3 localization (Supplementary Fig. 2b), indicating that these mutations do not perturb Sky1 kinase activity. These results clearly indicate that the reduction in stress granule localization upon PrLD deletion is due to the loss of the PrLD per se, rather than conformational changes in the protein resulting from deletions in the spacer region.

In addition, the PrLD was able to support stress-induced assembly when transferred to another protein. When expressed as a GFP fusion from a moderate-strength constitutive promoter, the PrLD was diffusely localized (Fig. 2c). Upon heat shock, it formed foci that colocalized with Pab1-mCherry foci (Fig. 2c). After 2 h recovery, the PrLD again showed almost entirely diffuse fluorescence (Fig. 2c), indicating that the PrLD is sufficient for reversible recruitment into stress granules.

**Fig. 3:**
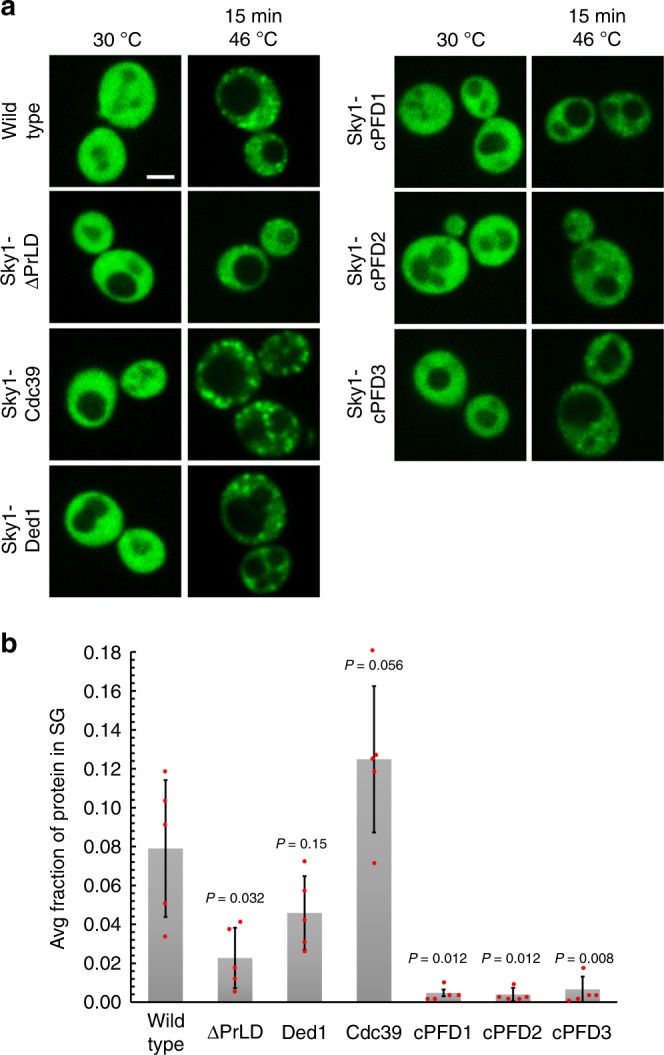
Other PrLDs can replace the Sky1 PrLD to support stress granule recruitment. **a** Yeast strains were generated expressing from the endogenous *SKY1* locus versions of Sky1-GFP in which the core PrLD was deleted (∆PrLD), replaced with the PrLDs from Cdc39 or Ded1, or replaced with nonaggregation-prone PrLDs (cPFD#1, cPFD#2, and cPFD#3). Cells were grown to mid-log, then subjected to heat shock at 46 °C for 15 min. Scale bar, 2 μm. **b** Quantification of the fraction of Sky1 in stress granule foci after 15 min of heat shock. Data represent mean ± SD of *n* = 5 independent replicates. A two-sided Mann–Whitney *U* test was used to calculate *P* values relative to wild-type. Source data are provided as a Source Data file

We next tested whether other PrLDs could substitute for the Sky1 PrLD in promoting stress granule recruitment. We previously showed that Cdc39 contains a PrLD that has similar compositional characteristics to the Sky1 PrLD, and that it efficiently forms foci when overexpressed in yeast^28^. Ded1 is a known stress granule protein, and contains a predicted PrLD^12,34^. When expressed as GFP fusions, the Cdc39 PrLD supported efficient stress granule recruitment (Supplementary Fig. 3a), while the Ded1 PrLD was weakly recruited into stress granules (Supplementary Fig. 3b). Consistent with this result, replacement of the Sky1 PrLD with the Cdc39 PrLD restored efficient recruitment to stress granules, while replacement with the Ded1 PrLD partially restored recruitment (Fig. 3a, b, Supplementary Fig. 3c, d).

Like most yeast prion domains, the Sky1 PrLD is glutamine/asparagine-rich. We previously built three synthetic PrLDs designed to have high glutamine/asparagine content, but low aggregation activity^28,34^. When expressed as GFP fusions, these control prion-forming domains (cPFDs) were not sufficient to support heat-induced assembly (Supplementary Fig. 3e). When the Sky1 PrLD was replaced with each of these cPFDs, stress-induced assembly was almost completely abolished (Fig. 3a, b). Thus, just as insertion of PrLDs that partition into granules supports Sky1 localization to granules upon heat stress, insertion of domains that localize to the cytoplasm upon heat stress actually increases cytoplasmic localization of Sky1 (Fig. 3b). The observed differences were not due to altered protein expression (Supplementary Fig. 3f). These experiments directly link the assembly activity of the PrLD to its ability to substitute for the Sky1 PrLD, and demonstrate that it is possible to block Sky1 recruitment by rationally modulating the assembly activity of the PrLD.

### Kinase-dependent disassembly of stress granules

**Fig. 4:**
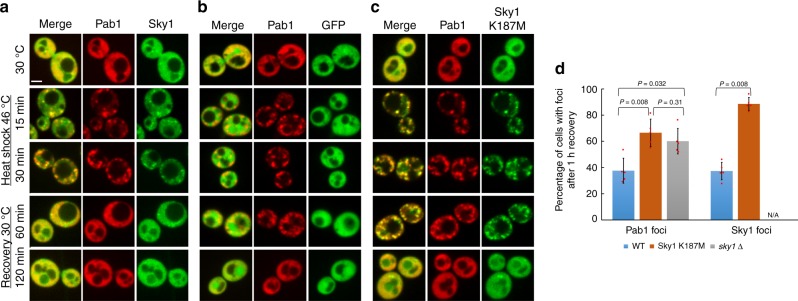
Sky1’s kinase activity is required for efficient stress granule dissolution. In yeast strains expressing Pab1-mCherry from the endogenous *PAB1* locus, the *SKY1* open reading frame was replaced with *SKY1*-GFP (**a**), the GFP open reading frame (**b**), or a catalytically inactive *sky1*^*K187M*^-GFP allele (**c**). Cells were grown to mid-log and visualized by fluorescence microscopy during heat shock and recovery. Scale bar, 2 μm. **d** Quantification of the percentage of cells containing Pab1 foci or Sky1 foci in wild-type (blue), *sky1*^*K187M*^ (orange), or ∆*sky1* (gray) cells. Data represent means ± SD of *n* = 5 independent replicates. A two-sided Mann–Whitney *U* test was used to calculate *P* values. Source data are provided as a Source Data file

Sky1 is an SR protein kinase that is responsible for regulating the phosphorylation state of nuclear shuttling proteins involved in mRNA export^32^. To test whether Sky1’s kinase activity might be involved in the regulation of stress granule formation or disassembly, we replaced the *SKY1* open reading frame with either the GFP open reading frame or a GFP-tagged *sky1*^*K187M*^ allele. Neither deletion nor inactivation of Sky1 prevented stress granule assembly (Fig. 4a–c and Supplementary Fig. 4a–b), indicating that Sky1 activity is not required for efficient stress granule formation. However, in both *sky1∆* and *sky1*^*K187M*^ strains, a significantly higher fraction of cells maintained Pab1-mCherry, Ded1-mCherry, and Pub1-mCherry foci after 1 h of recovery than in wild-type *SKY1* cells (Fig. 4a–d and Supplementary Fig. 4c). These data indicate Sky1’s kinase activity promotes efficient clearance of heat-induced stress granules. This effect may be in part by altering granule composition. Although Sky1 inactivation did not detectably affect the rate of Pab1 recruitment into granules, Sky1^K187M^ was more efficiently recruited to stress granules than wild-type Sky1 (Supplementary Fig. 4d), despite similar expression levels (Supplementary Fig. 4e).

Interestingly, while deletion of the Sky1 PrLD reduced recruitment to granules (Fig. 2), it did not significantly affect granule dissolution (Supplementary Fig. 5). This suggests either that recruitment to granules is not required for Sky1’s granule dissolution activity, or that the amount of Sky^∆PrLD^ recruited to granules is sufficient for its dissolution-promoting activity.

The slowed dissolution of granules in *sky1*^*K187M*^ strains also slowed recovery from stress. *sky1*^*K187M*^ strains did not show noticeable delays in resumption of translation after heat shock (Supplementary Fig. 6a–c), consistent with previous work showing that effects on stress granule disassembly are not strictly correlated with effects on translational recovery^11^; however, the *sky1*^*K187M*^ mutation did create a fitness cost for cells recovering from stress (Supplementary Fig. 6d). In the absence of heat shock, *sky1*^*K187M*^ strains grew slightly slower than wild-type cells, with average doubling times of about 101 min for *sky1*^*K187M*^ cells, versus 95 min for wild-type; therefore, after 7.5 h growth at 30 °C, wild-type cells achieved an ~24% higher OD than *sky1*^*K187M*^ cells. Heat shock accentuated this difference; when cells were grown for 30 min at 46 °C and then allowed to recover at 30 °C, wild-type cells achieved on average 55% higher OD than *sky1*^*K187M*^ cells after 7.5 h.

Furthermore, the effect of Sky1 inactivation was not specific to heat stress. Yeast cells maintained in stationary phase form stress granules^35^. Sky1 and Pab1 both form foci in stationary-phase cells (Supplementary Fig. 7a–c); although Sky1 and Pab1 foci showed less colocalization than under heat stress, ~19 ± 10% (mean ± SD of *n* = 5 independent replicates) of wild-type Sky1 and 30 ± 5% of Sky1^K187M^ colocalized with Pab1 foci. Sky1 foci were also largely separate from foci formed by Ded1, Pub1, and Tif4631 (Supplementary Fig. 7d–f). Despite this modest colocalization, inactivation of Sky1 nevertheless resulted in a higher fraction of cells showing Pab1 foci after 5 days in culture, as well as a higher fraction of cells with Pab1 foci after 30 min recovery (Supplementary Fig. 7). After 90 min of recovery, the differences between *SKY1* and *sky1*^*K187M*^ cells were no longer significant.

### Npl3’s phosphorylation state affects stress granules

We next examined the mechanism by which Sky1’s kinase activity influences stress granule dynamics. Sky1 has two confirmed in vivo targets, Npl3 and Gbp2^19,20^, as well as 40 additional potential targets identified by in vitro protein array experiments^21^. Npl3 seemed like a particularly intriguing candidate. Npl3 is a nucleocytoplasmic mRNA shuttling protein^36^ whose shuttling and mRNA dissociation is regulated by Sky1^31^. Upon heat stress, Npl3 shows increased cytoplasmic localization^37^, and Npl3 has been isolated from several different types of stress granules^18,24^. In addition, prolonged association of Npl3 with mRNA inhibits translation^38^. Finally, various core stress granule proteins, including Pab1, co-purify with TAP-tagged Npl3^39^.

To examine whether Sky1 inactivation affects this association between Npl3 and Pab1, cell extracts from cells expressing Npl3-GFP, Pab1-mCherry, and either wild-type Sky1 or Sky1^K187M^ were subjected to immunoprecipitation with anti-GFP nanobodies. Co-immunoprecipitation of Pab1 with Npl3 was modestly enhanced in the Sky1^K187M^ strain (Supplementary Fig. 8). This co-immunoprecipitation was almost completely lost upon RNase treatment, suggesting that Pab1 and Npl3 are in an RNA–protein complex. Thus, Sky1 inactivation increases association of Npl3 with two known stress granule components: RNA^31^, through a direct interaction, and Pab1, through an indirect association, likely bridged by RNA (Supplementary Fig. 8), making Npl3 a reasonable candidate for affecting granule dynamics.

**Fig. 5:**
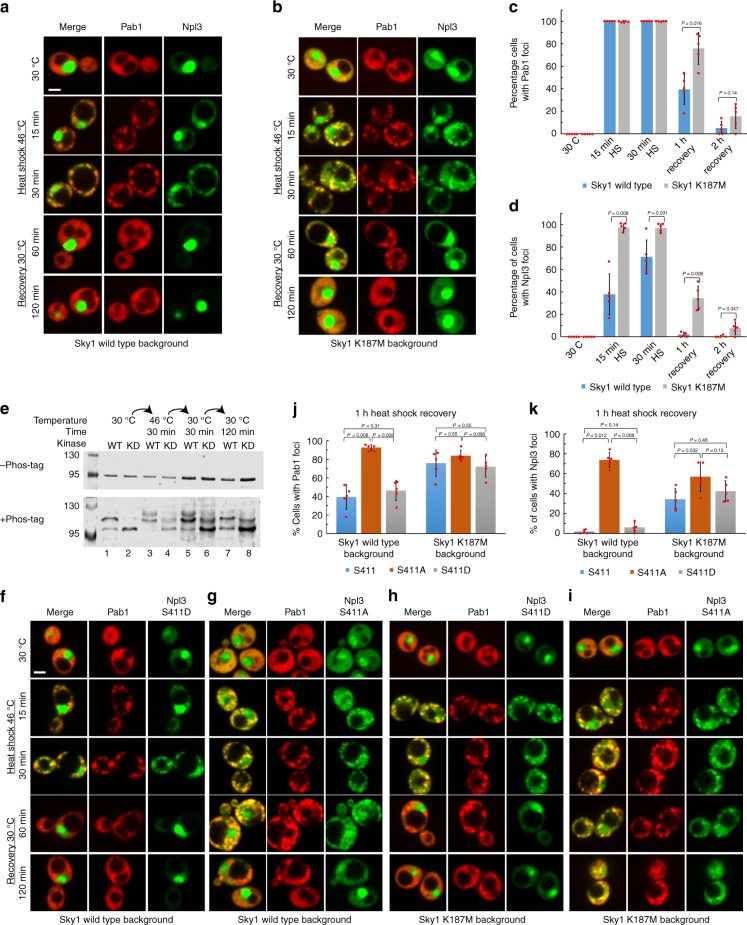
Npl3 phosphorylation by Sky1 is required for efficient stress granule dissolution. Fluorescence microscopy during heat shock and recovery of yeast expressing Pab1-mCherry, Npl3-GFP, and either wild-type Sky1 (**a**) or Sky1^K187M^ (**b**), each from its corresponding endogenous locus. Scale bar, 2 μm. Quantification of the percentage of cells containing Pab1 foci (**c**) or Npl3 foci (**d**) in wild-type *SKY1* (blue) or *sky1*^*K187M*^ (gray) cells. Data represent means ± SD of *n* = 5 independent replicates. A two-sided Mann–Whitney *U* test was used to calculate *P* values. **e** Yeast expressing Npl3-mCherry-His_8_ and either wild-type Sky1 (WT, odd lanes) or Sky1^K187M^ (Kinase Dead (KD), even lanes) from the corresponding endogenous loci were grown to mid-log phase in YPD, and subjected to a heat shock and recovery. Cells were analyzed by western blotting with an anti-His_6_ antibody, with and without the addition of Phos-tag. Fluorescence microscopy during heat shock and recovery of yeast expressing Pab1-mCherry; either Npl3^S411D^-GFP (**f**, **h**) or Npl3^S411A^-GFP (**g**, **i**); and either wild-type Sky1 (**f**, **g**) or Sky1^K187M^ (**h, i**) from the corresponding endogenous loci. Scale bar, 2 μm. Quantification of the percentage of cells containing Pab1 foci (**j**) or Npl3 foci (**k**) for cells from **a**, **b**, and **f**–**i** expressing wild-type Npl3 (blue), Npl3^S411A^ (orange)*,* or *NPL3*^*S411D*^ (gray). Data represent means ± SD of *n* = 5 independent replicates. A two-sided Mann–Whitney *U* test was used to calculate *P* values. Source data are provided as a Source Data file

We therefore characterized Npl3 protein localization during heat shock and recovery. As previously reported^36^, Npl3-GFP was predominantly nuclear under nonstress conditions (Fig. 5a). After 15 min of heat shock, Npl3-GFP was still predominantly nuclear, but showed a small increase in cytoplasmic localization (Fig. 5a). However, by 30 min of heat shock, in most cells Npl3-GFP had relocalized into cytoplasmic foci that colocalized with Pab1-mCherry (Fig. 5a). After 1 h of recovery, Npl3-GFP returned to the nucleus. Thus, Npl3-GFP is recruited into stress granules, but exhibits slower recruitment and more rapid disassembly dynamics than core stress granule proteins. In a *sky1*^*K187M*^ strain, Npl3-GFP was still predominantly nuclear in the absence of stress, but showed increased cytoplasmic localization (Fig. 5b). Upon heat stress, Npl3-GFP showed significantly faster relocalization to stress granules in the *sky1*^*K187M*^ strain, and these granules persisted significantly longer than in the *SKY1* strain (Fig. 5a–d). Thus, Sky1’s kinase activity regulates Npl3 localization during and after stress.

To confirm that Sky1 phosphorylates Npl3 during and after heat shock, we used phos-tag SDS-PAGE western blots to examine the phosphorylation profile of Npl3. Phos-tag is a compound that slows the electrophoretic migration of phosphorylated proteins^40^. Npl3 from both *SKY1* and *sky1*^*K187M*^ strains harvested under steady state conditions migrated predominantly as a single band in the absence of phos-tag (Fig. 5e). However, in the presence of phos-tag, Npl3 from the *SKY1* strain migrated as two bands; this second band was lost in the *sky1*^*K187M*^ strain (Fig. 5e lanes 1, 2), confirming that Npl3 shows Sky1-dependent phosphorylation, as previously reported^32^. The pattern was more complex during heat shock and recovery. Under both conditions, only a single band was observed by western blot in the absence of phos-tag for both the *SKY1* and *sky1*^*K187M*^ strains (Fig. 5e). For the *SKY1* strain, addition of phos-tag resulted in the appearance of two slower-migrating bands, only one of which was lost in the *sky1*^*K187M*^ strain (Fig. 5e, lanes 3–6). This indicates that during and after heat stress, Npl3 is subject to both Sky1-dependent and Sky1-independent phosphorylation. Sky1 is reported to phosphorylate Npl3 at a single site, S411^31^. To confirm that the Sky1-dependent changes observed on the phos-tag gel were a result of phosphorylation at this site, the experiment was repeated in an *npl3*^*S411A*^ strain. Under all conditions, Npl3^S411A^ lost the slower-migrating *SKY1-*dependent bands that were seen on the phos-tag gel for wild-type Npl3, confirming that Sky1 phosphorylates Npl3 before, during, and after heat shock (Supplementary Fig. 9).

To determine whether this Sky1-dependent phosphorylation of Npl3 is responsible for Sky1’s effects on stress granule dynamics, we replaced *NPL3* with alleles encoding either the nonphosphorylatable mutant Npl3^S411A^ or the phosphomimetic mutant Npl3^S411D^. Although Pab1 rapidly formed foci in all strains upon heat stress (Fig. 5a, b, f–i and Supplementary Fig. 10a), both Npl3 and Pab1 showed more efficient recruitment into Pab1 foci in the *npl3*^*S411D*^ and *npl3*^*S411A*^ strains than in the *NPL3* strain (Supplementary Fig. 10b–d). However, only the *npl3*^*S411A*^ mutation mimicked the effects of *sky1*^*K187M*^ stress granule dissolution, with both Pab1 and Npl3 foci persisting significantly longer in the *npl3*^*S411A*^ strain than in either the *NPL3* or *npl3*^*S411D*^ strains (Fig. 5f–k). Interestingly, Sky1 also exerted an effect on granule dynamics that was independent of Npl3 phosphorylation at S411, as the *sky1*^*K187M*^ *npl3*^*S411D*^ double mutant showed slower dissolution of Pab1 foci than the *npl3*^*S411D*^ strain (Fig. 5f, h, j; *P* = 0.023).

### Sky1 activity can compensate for Hsp104 deficiencies

**Fig. 6:**
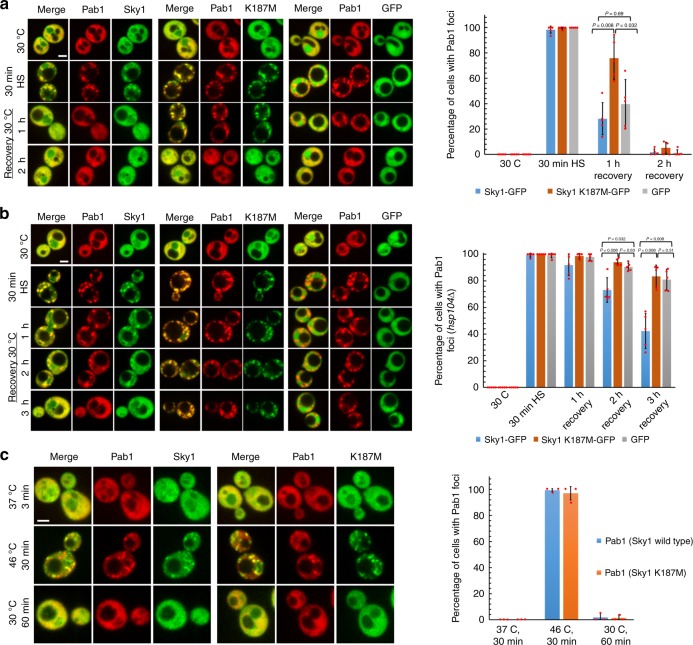
Sky1 overexpression can compensate for defects in other stress granule dissolution pathways. Either an *HSP104* (**a**) *or hsp104∆* (**b**) strain expressing Pab1-mCherry from its endogenous locus was transformed with plasmids expressing Sky1-GFP (blue bars), Sky1^K187M^-GFP (orange bars), and GFP (gray bars) under control of the *GPD* promoter. Cells were grown in synthetic dropout medium and visualized by fluorescence microscopy during a heat shock and recovery; the percentage of cells containing Pab1 foci was determined. Scale bar, 2 μm. Data represent means ± SD of *n* = 5 independent replicates. A two-sided Mann–Whitney *U* test was used to calculate *P* values. **c** Pretreatment with mild heat stress to upregulate chaperone levels compensates for *sky1* deficiency. Yeast expressing Pab1-mCherry and either Sky1-GFP (blue bars) or Sky1^K187M^-GFP (orange bars) from the corresponding endogenous loci were grown to mid-log phase, and then subjected to 37 °C for 30 min to upregulate chaperone levels. Scale bar, 2 μm. Data represent means ± SD of *n* = 3 independent replicates. Source data are provided as a Source Data file

Various yeast chaperones, including Hsp104, Hsp70s, and Hsp40s are required for efficient stress granule clearance^10,11^. We therefore asked whether increasing Sky1 activity could compensate for loss of chaperones that are required for efficient stress granule disassembly. Plasmids expressing GFP, Sky1-GFP, and Sky1^K187M^-GFP from the strong constitutive *GPD* promoter were introduced into either an *hsp104∆* or *HSP104* strain containing the wild-type *SKY1* gene. In the presence of *HSP104*, overexpression of Sky1-GFP had little effect on formation or dissolution of Pab1-mCherry foci, while overexpression of Sky1^K187M^-GFP exerted a dominant-negative effect, slowing dissolution of Pab1-mCherry foci even in the presence of an endogenous copy of *SKY1* (Fig. 6a). As expected, the *hsp104∆* strain showed substantially slower dissolution of Pab1-mCherry foci. Overexpression of Sky1-GFP, but not Sky1^K187M^-GFP significantly accelerated dissolution of foci (Fig. 6b), indicating that elevated Sky1 kinase activity can partially reverse the effect of chaperone deficiencies.

We likewise asked whether elevated chaperone levels could compensate for deficiencies in Sky1 activity. Pretreatment of yeast cells at 37 °C elevates chaperone levels, and increases survival during heat shock at 46 °C^41^. Pretreatment of both *SKY1* or *sky1*^*K187M*^ strains at 37 °C for 30 min prior to 46 °C heat shock substantially accelerated the dissolution of Pab1-Cherry foci during recovery from heat stress, with almost complete elimination of visible foci after 60 min of recovery (Fig. 6c). These results highlight the extent to which multiple pathways contribute to stress granule dissolution, and demonstrate that it is possible to compensate for deficiencies in one pathway by upregulating proteins in other pathways.

## Discussion

When cells are faced with fluctuating environments, they utilize elaborate mechanisms to spatiotemporally reorganize cellular processes for cellular adaptation and survival. Central to this cellular response is the rapid formation of stress granules, which are dynamic cytoplasmic RNA–protein assemblies. Importantly, once the cellular stress is relieved, stress granules rapidly dissolve, allowing the cell to regain the normal cellular functions that were repressed during stress. The dysregulation of stress granules has detrimental consequences for the cell and can lead to neurodegenerative diseases^3^, but the intricate mechanisms underlying the regulation of stress granules are not fully understood. Here, we identify Sky1 as a regulator of stress granule disassembly, and demonstrated that this activity is mediated in part by phosphorylation of an RNA-shuttling protein, Npl3.

PrLDs are prevalent in stress granule proteins, and mutations in various PrLD-containing RNA-binding proteins have been linked to degenerative diseases^3,42,43^. Disease-causing mutations are associated with the formation of cytoplasmic inclusions that share common components with stress granules^3,42,43^. In vitro, many disease-associated mutations accelerate conversion to a stable amyloid state, or perturb LLPS dynamics^44–48^. These results have led to the hypothesis that these PrLDs have evolved to mediate LLPS interactions that help localize proteins to granules, and that disease-associated mutations dysregulate granule formation and disassembly, or convert granules to more stable structures^3,42,43^.

PrLDs’ ability to self-assemble may help cells respond to fluctuations in the environment. Moreover, PrLDs provide conformational flexibility to participate in weak noncovalent multivalent interactions. Therefore, deleting the PrLDs would decrease the number of interactions available, leading to a reduction in stress granule formation^29,49^. Interestingly, although PrLD deletion reduced Sky1’s localization to stress granules, it did not detectably affect Sky1’s granule dissolution activity. However, it remains possible that PrLD deletion has effects on disassembly under specific conditions, or that were below our threshold of detection. Importantly, although a number of studies support a role for PrLDs in stress granule recruitment, our work builds on these studies by rigorously testing predictions of this model. First, our work directly links the assembly activity of the PrLDs to recruitment, demonstrating that localization to granules can be increased or decreased through the addition of PrLDs that preferentially partition into either stress granules or the cytoplasm, respectively. Second, we show that the Sky1 PrLD acts in a modular, position-independent manner to support stress granule recruitment, and that the ability of other PrLDs to substitute for the Sky1 PrLD in supporting recruitment is directly related to their assembly activity.

An obvious question is why Sky1 had not been previously identified as a stress granule component. Various studies have attempted to characterize the diversity of yeast proteins sequestered in stress granules^18,22–25^. However, other known regulators of stress granules, including Hsp104, Ydj1, and Sis1, have not always been identified by these methods^18,23^. In addition, while Sky1 is enriched in granules, this enrichment is less dramatic than for other stress granule markers (Fig. 1a, Supplementary Fig. 1a), potentially explaining why it was not detected. This failure to identify regulators may also reflect limitations of the methods used to characterize stress granule proteins. In most of these studies, stress granules were first isolated from cells, allowing for proteomic identification of the components. Components that are weakly associated with stress granules may be lost during the isolation of stress granules. Consistent with this explanation, recent studies using proximity labeling have identified Sky1’s human homolog, SRPK1^50^, and Npl3’s human homolog, SRSF1^51^, as being associated with stress granules.

Posttranslational modifications, including protein phosphorylation, are postulated to broadly regulate aggregation-prone proteins found in stress granules^1,14,15^, and several studies have shown that protein phosphorylation can regulate aggregation propensity in vivo and in vitro^52–54^. However, the specific kinases and phosphatases involved in stress granule regulation, and the targets of these enzymes, have not been fully delineated.

While our results clearly suggest that Sky1-dependent phosphorylation of Npl3 affects stress granule dynamics, the observation that Sky1 inactivation delays granule dissolution even in an *npl3*^*S411D*^ strain suggests that Sky1 may be acting on other targets to influence granule dissolution. The full range of Sky1 targets is not known. Sky1 is the only SR protein kinase in yeast. In mammals, SR kinases generally phosphorylate regions with multiple SR dipeptide repeats.^32^ However, the two confirmed in vivo targets of Sky1, Npl3, and Gbp2^19,20^, lack extended SR repeats. Instead, Npl3 is phosphorylated at an isolated RS dipeptide, while Gbp2 is phosphorylated in a short stretch of three RS dipeptides^48^. Thus, Sky1 may phosphorylate other proteins containing single SR dipeptides or short SR dipeptide motifs. In fact, protein mircoarray experiments have identified 40 proteins that can be phosphorylated by Sky1 *in vitro*^21^.

While the effects of Sky1 inactivation were most obvious for granule dissolution, it also affected granule formation. Npl3 is more rapidly recruited to foci upon heat stress in a *sky1*^*K187M*^ strain, perhaps because Sky1 inactivation increases the concentration of Npl3 in the cytoplasm, thereby facilitating its rapid recruitment into granules. Likewise, Sky1 accumulates in granules to a greater degree upon heat stress in a *sky1*^*K187M*^ strain, possibly due to its interaction with Npl3 or other stress granule components. The effect on stress granule assembly was even more striking during extended stationary phase, where Sky1 inactivation resulted in a statistically significant increase in the fraction of cells with stress granules (Supplementary Fig. 7). It is therefore possible that the Sky1 inactivation affects the structure, composition, or stability of granules, or protein levels of granule components, and that these changes contribute to the observed effects on granule dissolution.

Although Npl3 has previously been identified in yeast^18,24^ and mammalian cells^24,51^ as a component of stress granules, its phosphorylation state has not previously been implicated in regulating stress granules dynamics. During steady state conditions, Npl3 binds mRNA in the nucleus to facilitate mRNA export. Phosphorylation by Sky1 in the cytoplasm reduces Npl3’s binding affinity to mRNA^31^, resulting in the release of RNA and rapid nuclear reimport of Npl3^20^. This suggests a few mechanisms by which Npl3 phosphorylation could impact stress granule dynamics. The simplest explanation is that phosphorylation may simply reduce the number of multivalent interactions available to Npl3. mRNA is present in stress granules and plays a major role as a scaffolding factor to stabilize multivalent interactions within granules^55–58^. Phosphorylation reduces Npl3’s mRNA binding^31^, and reduces its RNA-dependent association with Pab1. Thus, enhancement of these direct and indirect interactions with stress granule components upon Sky1 inactivation may stabilize the network of interactions within granules. It should be noted that while one previous study suggests Npl3 is preferentially exported from the nucleus without RNA during stress, these experiments were performed under mild heat shock (42 °C) for 15 min^37^. We showed Npl3 is not recruited to stress granules until severe stress (46 °C) for 30 min (Fig. 5a, b). Alternatively, because prolonged association of Npl3 with RNA inhibits translation^38^, and because Npl3 can directly interact with eIF4G to repress translation^59^, phosphorylation of Npl3 may promote stress granule dissolution by releasing stalled translation complexes. Although blocking Npl3 phosphorylation did not detectably affect resumption of translation after heat stress, it remains possible that blocking Npl3 phosphorylation delays resumption of translation for subset of mRNAs. Finally, Npl3’s shuttling activity may contribute to stress granule dissolution. Nuclear–cytoplasmic shuttling mechanisms are closely linked to mammalian stress granule regulation, and defects have recently been linked to the progression of neurodegenerative disease in mammalian cells^60–62^.

In summary, we have demonstrated that Sky1-mediated regulation provides a pathway for dissolution of stress granules. During stress recovery, a concerted process between molecular chaperones, RNA helicases, and Sky1 actively targets granules for efficient dissolution, allowing cellular components sequestered in stress granules during stress to regain normal function. Intriguingly, these pathways are clearly partially redundant, as Sky1 overexpression could partially compensate for *hsp104* deletion, and stimulating chaperones by pretreatment with mild heat stress could partially compensate for Sky1 inactivation. This result has significant potential health implications. If SRPK1 has a similar function in mammalian cells, targeted increase in SRPK1 may be able to compensate for disease-associated defects in the disassembly machinery.

## Methods

### Yeast strains and media

Yeast strains were grown at 30 °C in YPD media (10 g L^−1^ yeast extract, 20 g L^−1^ peptone, and 20 g L^−1^ glucose), unless otherwise indicated. See Supplementary Table 1 for strain descriptions, Supplementary Table 2 for plasmids, and Supplementary Table 3 for primers used for strain construction. All strains and plasmids are available upon request.

A PCR-based homologous recombination protocol based on the methods of Longtine et al.1998^63^ was used to endogenously tag genes with mCherry. Briefly, an mCherry-URA3 cassette was PCR amplified from pRS306-mCherry (a kind gift from Dr Steven Markus), adding regions of homology to allow for in-frame fusion of the cherry tag with the target gene. These products were transformed into the Sky1-GFP strain from the GFP collection^64^, selecting on SC-Ura. Genomic PCR and DNA sequencing were performed to confirm correct insertion.

A flip in, flip out scheme was used to generate endogenous Sky1 mutants, adapted from Riback et al.^65^ First, a URA3 cassette was inserted into the target site within *SKY1* using homologous recombination. Next, PCR products were generated containing the desired mutations; these PCR products were transformed into the *URA3* strain. Cells were plated on medium containing 5-FOA to select for loss of the URA3 cassette, and mutations were confirmed using genomic PCR and DNA sequencing.

To generate Npl3 (S411A/S411D)-GFP strains for microscopy, the *HIS3* cassette from pFA6a-GFP(S65T)-HIS3MX6^63^ was PCR amplified, adding regions of homology to allow for in-frame fusion of the GFP tag, while changing the S411 codon. This fragment was inserted into Pab1-mCherry strains by homologous recombination.

To generate Npl3-mCherry-His_8_ strains for phos-tag western blots, the KanMX cassette from pFA6a-GFP(S65T)-KanMX6^63^ was PCR amplified, adding a His_8_-tag at the 5′end. This fragment was inserted into Npl3-mCherry strains by homologous recombination. To generate the *hsp104Δ* strain, a *HIS3* cassette was amplified from pRS313^28^, and inserted in place of the *HSP104* open reading frame in strain yER1171 by homologous recombination. All transformants were confirmed by PCR and DNA sequencing. See Supplementary Table 3 for primers used for strain construction.

### Plasmid construction

To generate the GFP-PrLD fusions, first GFP was cloned under control of the *SUP35* promoter by replacing the Sup35 open reading frame in pJ526^66^, adding a GSAGGS spacer, and BamHI and BglII sites, to generate plasmid pER843. Sky1’s PrLD was amplified from the strain ATCC 201388^64^ and cPFD1-3 were amplified from plasmids pER1965, pER1947, and pER1948^28^, adding BglII and BamHI restriction sites. PCR products were cut with BglII and BamHI and cloned into pER843 to generate GFP-PrLDs fusions expressed from the *SUP35* promoter.

To create Sky1-GFP fusions under control of the strong constitutive *GPD* promoter, Sky1-GFP, Sky1 K187M-GFP, and GFP open reading frames were amplified from yER1001 and yER1530, adding tails homologous to pAG415GPD-ccdB^67^ (Addgene plasmid # 14146; from Susan Lindquist) for In-fusion cloning. All plasmids were confirmed by PCR and DNA sequencing. See Supplementary Table 2 for plasmid descriptions, and Supplementary Table 4 for primers used for plasmid construction.

### Microscopy

To monitor stress granule formation, yeast strains were grown to mid-log phase in YPD. One milliliter of cell culture was harvested and washed in SC media prior to imaging by confocal microscopy using an Olympus IX81 Inverted Spinning Disk Microscope and a cascade II EMCCD camera. Heat shock and recovery were performed in 46 °C and 30 °C water baths, respectively. To upregulate chaperone levels, cells were incubated at 37 °C for 30 min prior to heat shock.

To monitor stress granule association by GFP-PrLD fusions, yeast strain yER1405 was transformed with *LEU2* plasmids expressing GFP-PrLD from the *SUP35* promoter. Cells were grown in dropout medium lacking leucine to mid-log phase, and analyzed as above.

### Quantification of microscopy images

To determine the percentage of cells with foci, at least 30 cells from at least three independent experiments were counted. A two-sided Mann–Whitney *U* test was used to determine statistical significance at 1 h recovery time points.

To determine colocalization of Sky1 foci with Pab1 foci, at least 30 cells from at least three independent experiments were analyzed. Quantification was performed using Slidebook (Intelligent Imaging Innovations, 3i). A two-dimensional Laplacian filter was applied to each channel. For both channels, average background was calculated for each cell, and pixels within the cell with fluorescence 50% or more above background we considered to be in foci. Colocalization was defined as the percentage pixel area overlap of green foci with red foci.

To determine the fraction of protein in stress granules, images of at least 30 cells from five independent experiments were analyzed. Quantification was performed using Slidebook (Intelligent Imaging Innovations, 3i). Region masks were applied to cells by tracing the cell outline. Then, a 2D Laplacian filter was applied, and the average background fluorescence for all cells was calculated. Regions within each cell with fluorescence 50% or more above background were considered granules. For each cell, the fraction of protein in granules was calculated as the fraction of total signal found within the stress granule mask. A two-sided Mann–Whitney test was used to determine statistical significance.

### Western blotting

To assess protein expression levels, Sky1-GFP strains were grown to mid-log phase. Cells were harvested and lysed in lysis buffer (2 M Urea, 10 mM EDTA, 10 mM MOPS, and pH 6.8). Samples were normalized based on total protein. The membrane was probed with an anti-GFP primary antibody (Santa Cruz; sc-9996, 1:1000), and Alexa Fluor IR800 goat anti-mouse secondary antibody (Rockland, 610-132-121; 1:50,000).

To monitor protein phosphorylation, we supplemented SDS-PAGE gels with 50 μM phos-tag^TM^ (Wako). Yeast strains were grown to mid-log phase in YPD, then cell cultures were incubated in a 46 °C water bath, followed by recovery at 30 °C for the indicated time. To halt all enzymatic activity, trichloroacetic acid (TCA) was directly added to cell cultures to a final 6%, and the mixture was incubated in an ice slurry bath for 10 min. Cells were harvested, lysed in 2 M lysis buffer, and normalized based on total protein. Normalized protein samples were TCA/acetone precipitated to remove contaminants and resuspended in 1× running buffer (15% glycerol, 80 mM Tris base, 3.5% SDS, bromophenol blue). Samples were run on 7.5% acrylamide SDS-PAGE gels and 7.5% acrylamide SDS-PAGE gels with 50 μM phos-tag and 100 μM MnCl_2_. To remove the Mn^+^ ions, the gel was incubated in 1× transfer buffer + 10 mM EDTA for 20 min twice, followed by 1x transfer buffer for 10 min. Protein was transferred to PVDF membrane. The membrane was probed with an anti-His Tag primary antibody (Sigma, SAB1305538; 1:500), and Alexa Fluor IR800 goat anti-mouse secondary antibody (1:50,000).

### Resumption of translation

Cells were grown to mid-log at 30 °C with shaking, and then subjected to 30 min heat shock at 46 °C, followed by recovery at 30 °C. For each strain, cells were harvested before and after heat shock, as well as after 45 min of recovery at 30 °C. Cells were pelleted by centrifugation (3000 rpm for 5 min at room temperature), washed once in SC media lacking methionine (SC-met), and then resuspended in 1 mL SC-met. l-homopropargylglycine (l-HPG; ThermoFisher), a methionine analog, was added to each culture at a final concentration of 50 µM and incubated for 10 min for incorporation into nascent proteins. Cells were harvested and lysed in 50 mM Tris HCl pH 8.0/1% SDS/1× ProBlock Gold Yeast Protease Inhibitor Cocktail (Gold Biotechnology). Alexa488 azide was conjugated via click chemistry to nascent proteins using a Click-iT Protein Reaction Buffer Kit (ThermoFisher), followed by methanol/chloroform precipitation, according to manufacturer recommendations. Proteins were resuspended in lysis buffer, vortexed for 5 min, heated at 70 °C for 10 min, and centrifuged at 11,000 rpm for 5 min. Samples were analyzed by polyacrylamide gel electrophoresis. Total protein and l-HPG-labeled protein were quantified using Image Studio Lite version5.2.

### Co-immunoprecipitations

Preparation for co-IP was performed using a protocol adapted from Feliciano et al.^68^ For each strain, overnight yeast cultures were diluted into 2 L cultures at a starting OD = 0.5, cultured for two doubling times, and harvested by centrifugation at 3150 × *g* for 20 min at 4 °C. Pellets were resuspended in 4 mL ice-cold ultra-pure H_2_O, flash frozen in liquid N_2_, and lysed by grinding/blending in liquid N_2_. Two hundred milligrams of powdered yeast were resuspended in 200 µL of IP buffer [10 mM Tris HCl pH7.5, 150 mM NaCl, 0.5 mM EDTA, 1x Protease Inhibitor cocktail (ProBlock Gold Yeast/Fungi Protease Inhibitor Cocktail, Gold Biotechnology), and 1x RiboLock RNase Inhibitor (Thermo Fisher Scientific) where applicable]. Cellular debris was cleared by centrifugation at 17,900 x *g* for 1 min. Npl3-GFP was immunoprecipitated on GFP-Trap magnetic beads (ChromoTek) according to manufacturer protocol. These beads contain a single variable domain nanobody. After the initial immunoprecipitation step, beads were washed once in ice-cold IP buffer, then resuspended in 250 µL IP buffer with or without RNase A and rotated for 15 min at room temperature. Beads were washed two additional times, and immunoprecipitated protein was eluted by boiling for 10 min in 2× SDS loading buffer. Total lysate and elution fractions were loaded onto 12.5% polyacrylamide gels with elution fractions 5× concentrated relative to the total lysate fractions, then transferred to PVDF membrane. The membrane was probed with either an anti-mCherry primary antibody (Millipore, MAB131973; 1:2,500) an anti-GFP primary antibody (Santa Cruz, sc-9996; 1:500), and Alexa Fluor IR800 goat anti-mouse secondary antibody (Rockland, 610-132-121; 1:50,000).

### Reporting Summary

Further information on research design is available in the Nature Research Reporting Summary linked to this article.

## Supplementary information

Supplementary Information

Reporting Summary

Source Data

## Data Availability

The data that support the findings of this study are available within the paper, Supplementary Information, or available from the corresponding author upon request. Source data underlying Figs. 1–6 and Supplementary Figs. 1–10, are provided as a Source Data file.
