# Supplementary material for: The prion-like protein kinase Sky1 is required for efficient stress granule disassembly
Source: Nat Commun. 2019 Aug 9;10:3614. doi: 10.1038/s41467-019-11550-w (PMC6688984; doi:10.1038/s41467-019-11550-w)
Supplement: Supplementary file 1 — Supplementary Information [file 41467_2019_11550_MOESM1_ESM.pdf]

The prion-like protein kinase Sky1 is required for efficient stress granule disassembly

Shattuck et al.

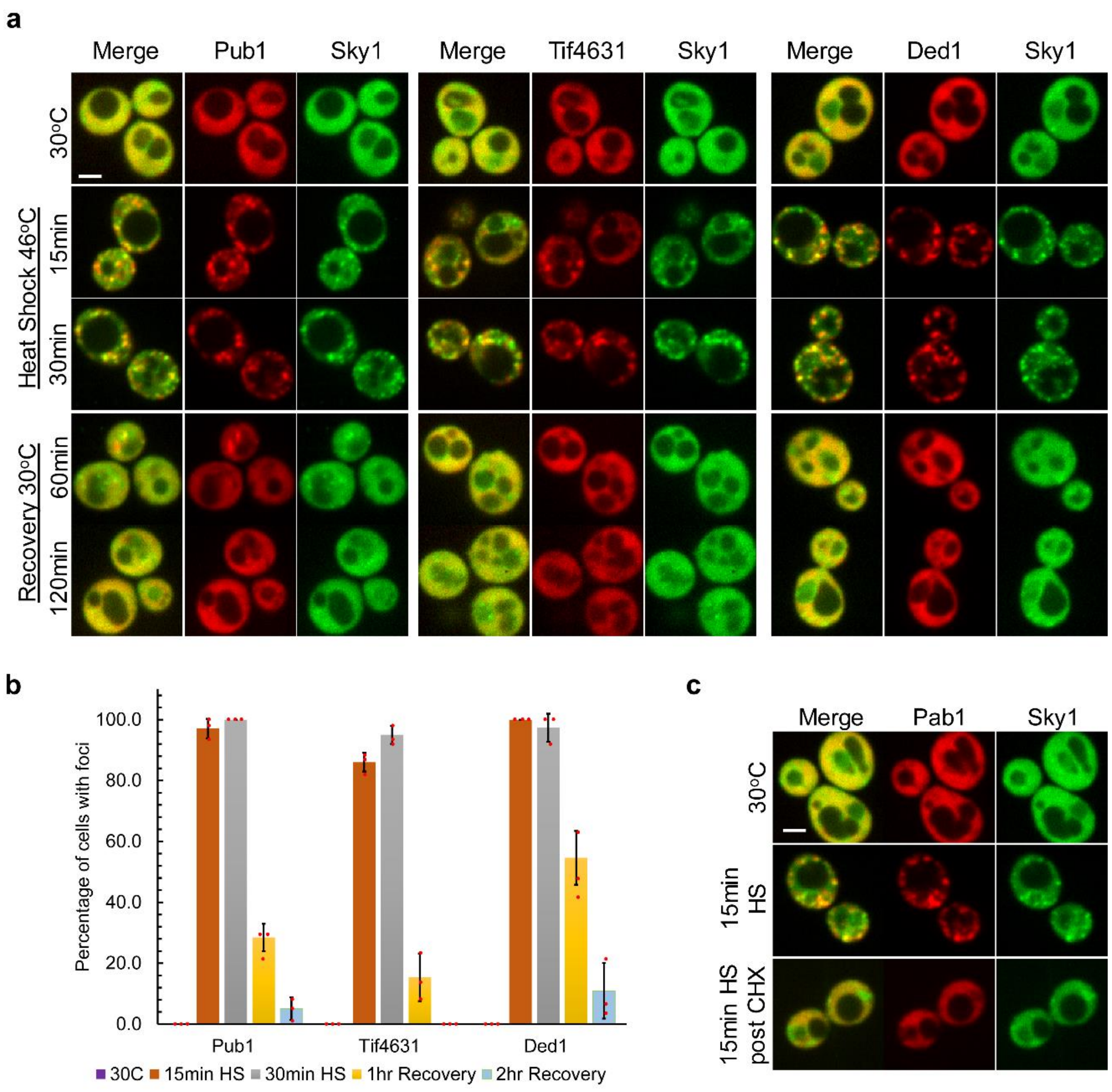

Supplementary Figure 1: Sky1 is a stress granule protein

(**a**) Sky1 colocalizes with multiple stress granule markers during heat stress. Yeast strains expressing Sky1-GFP and the known stress granule proteins Pub1-mCherry, Tif4631-mCherry, or Ded1-mCherry, each from their corresponding endogenous locus were grown to mid log phase and visualized by fluorescence microscopy during heat stress and recovery. Scale bar, 2 µm. (**b**) Quantification of the percentage of cells with foci prior to heat shock (30C, purple bars); after 15 min (orange) and 30 min (grey) heat shock; and after 1 hr (yellow) and 2 hr (blue) recovery. Data represent means $\pm$ SD of 3 independent experiments. (**c**) Pre-treatment of cells with cycloheximide reduces heat-induced foci formation by Sky1. Yeast expressing Sky1-GFP and Pab1-mCherry from their corresponding endogenous loci were grown to mid-log. Cells were then either subjected to heat shock for 15 minutes at 46ºC, or pre-treated with 50ug/ml cycloheximide for 10 minutes prior to heat shock. Scale bar, 2 µm. Source data are provided as a Source Data file.

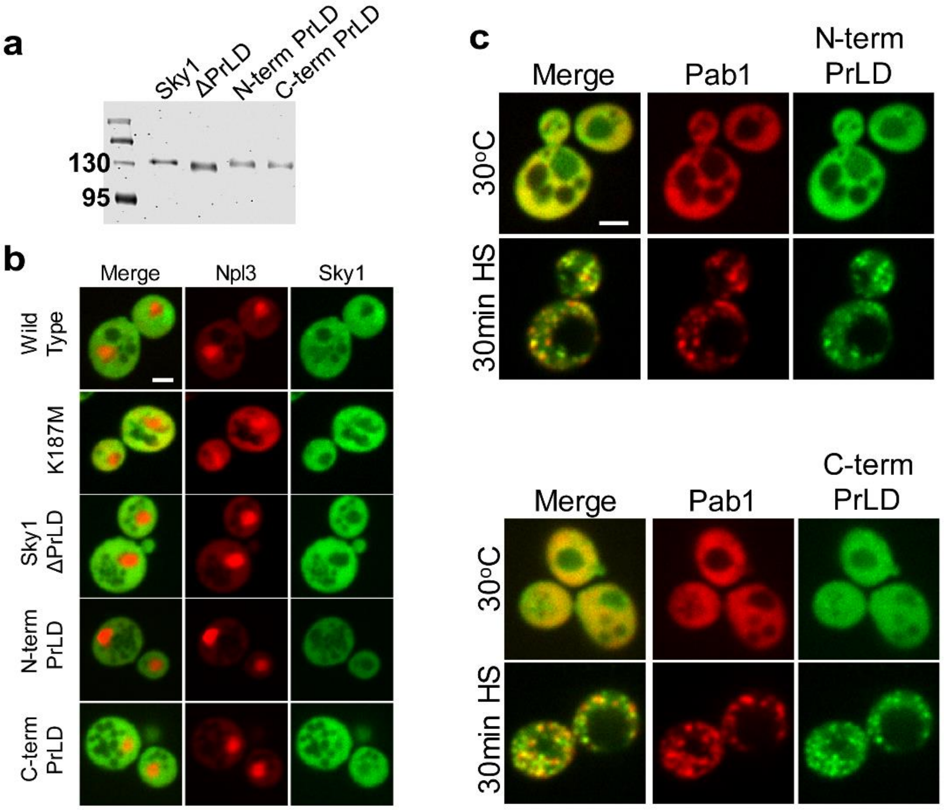

Supplementary Figure 2: Sky1 PrLD mutant constructs are functional.

(**a**) Western blot of PrLD rearrangement mutants. (**b**) Deleting or moving the Sky1 PrLD does not substantially alter Sky1-dependent localization of Npl3. Fluorescence microscopy of yeast expressing from the corresponding endogenous loci Npl3-mCherry and GFP-tagged Sky1 mutants in which the core PrLD was deleted (ΔPrLD) or moved to the N-terminus (N-term PrLD) or C-terminus (C-term PrLD). Scale bar, 2 μm. (**c**) Sky1 mutant constructs localize to stress granules. Yeast expressing Sky1-GFP mutants and Pab1-mCherry were visualized by fluorescence microscopy during heat shock. Scale bar, 2 μm. Source data are provided as a Source Data file.

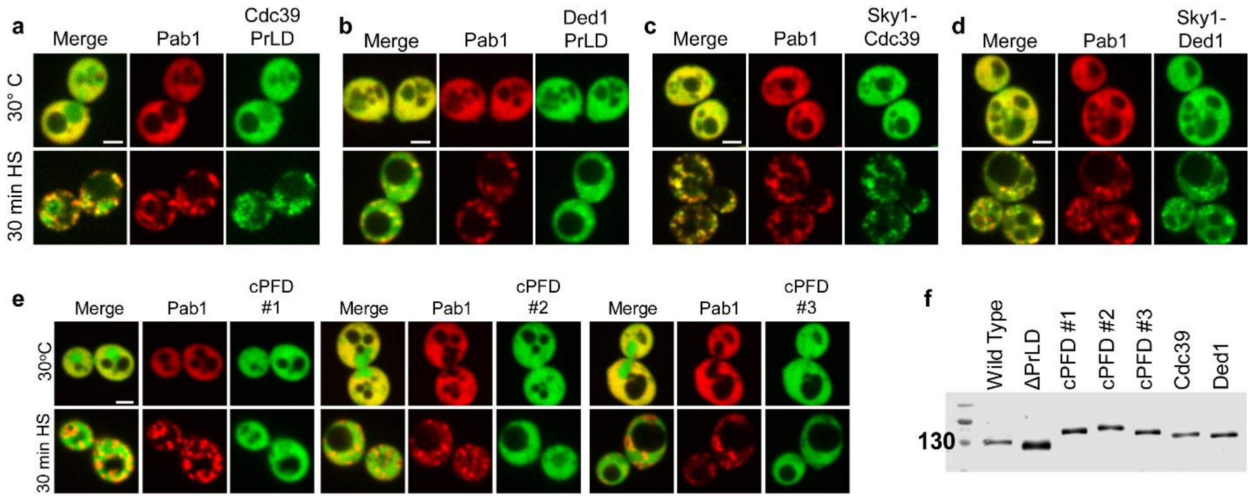

Supplementary Figure 3: cPFDs are not sufficient to be recruited to stress granules.

(**a,b**) The Cdc39 and Ded1 PrLDs are recruited to stress granules. Yeast expressing Pab1-mCherry from its endogenous locus were transformed with plasmids expressing GFP-tagged versions of the Cdc39 or Ded1 PrLD under control of the *SUP35* promoter. Cells were grown to mid-log and visualized by fluorescence microscopy during a heat shock. Scale bar, 2 µm. (**c,d**) Sky1 mutants in which the PrLD was replaced with the Cdc39 or Ded1 PrLD are recruited to stress granules. Scale bar, 2 µm. (**e**) The cPFDs are not recruited into stress granules. Scale bar, 2 µm. (f) Western blot analysis with an anti-GFP antibody of mutants in which the Sky1 PrLD was deleted or replaced. Source data are provided as a Source Data file.

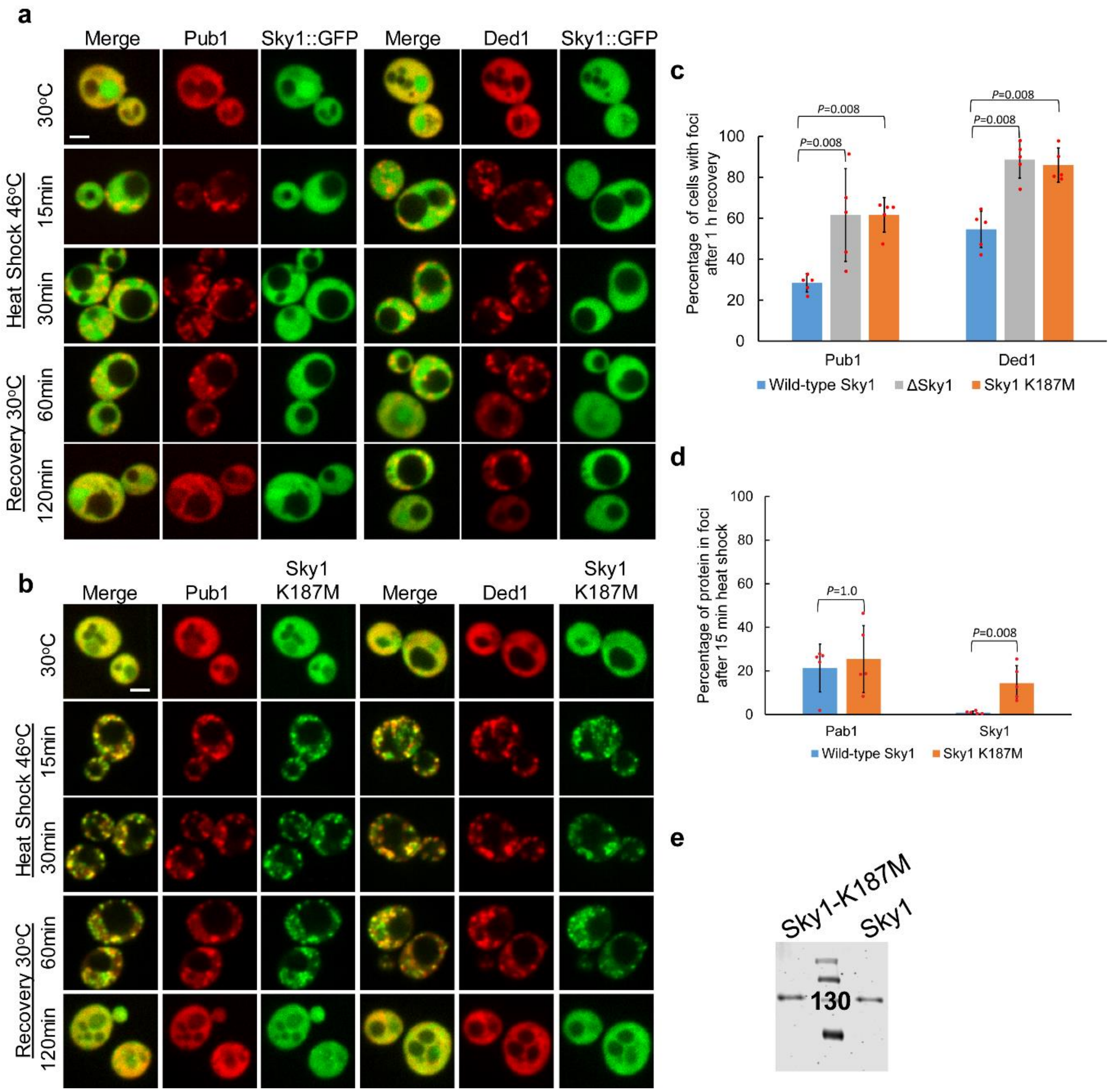

Supplementary Figure 4: Sky1 and its kinase activity is required for efficient dissolution of stress granules.

(**a-b**) In yeast strains expressing mCherry-tagged granule proteins from the corresponding endogenous loci, the *SKY1* open reading frame was replaced with either the GFP open reading frame (a) or a catalytically inactive *sky1*^{K187M}-GFP allele (**b**). Cells were visualized by fluorescence microscopy during heat shock and recovery. Scale bar, 2 µm. (**c**) Quantification of the fraction of cells with foci in wild-type (blue), Δ*sky1* (grey), or *sky1*^{K187M} (orange) cells after 1 h recovery. Data represent means ± SD of 5 independent experiments. A two-sided Mann-Whitney *U* test was used to calculate *P* values. (**d**) Quantification of the percentage of Pab1 and Sky1 in foci in wild-type (blue) or *sky1*^{K187M} (orange) cells after 15 min heat shock. Data represent means ± SD of 5 independent experiments. A two-sided Mann-Whitney *U* test was used to calculate *P* values. (e) Western blot analysis with an anti-GFP antibody, comparing expression of Sky1-GFP and Sky1^{K187M}-GFP.

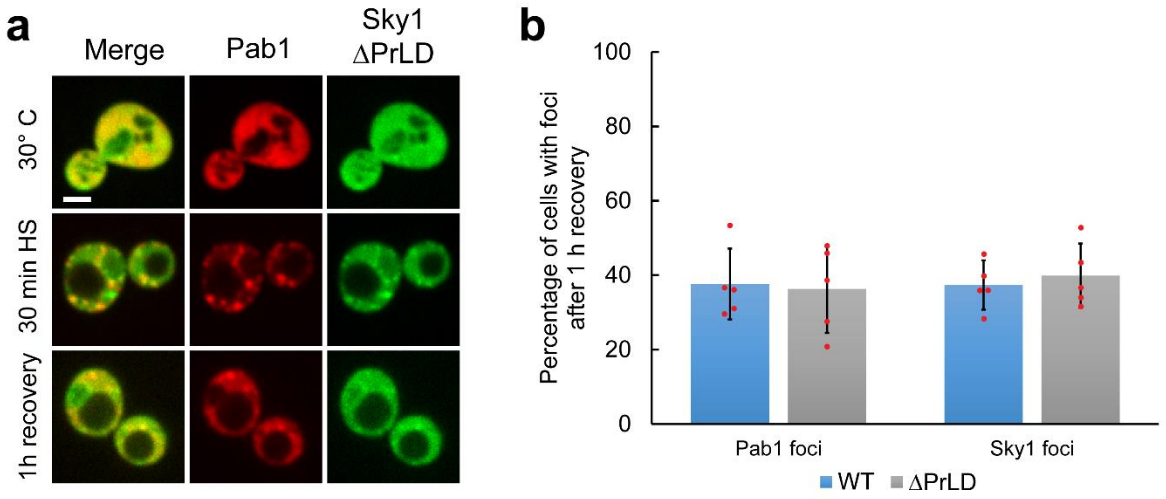

Supplementary Figure 5: Deletion of the Sky1 PrLD does not significantly affect stress granule dissolution.

(**a**) Yeast expressing Sky1$^{\Delta PrLD}$-GFP and Pab1-mCherry from the corresponding endogenous locus were grown at 30°C to mid-log, subjected to heat shock at 46°C, and allowed to recover at 30°C. Cells were visualized by fluorescence microscopy during heat shock and recovery. Scale bar, 2 μm. (**b**) Quantification of the percentage of cells with Sky1 or Pab1 foci for cells expressing wild-type Sky1 (blue) or Sky1$^{\Delta PrLD}$-GFP (grey). Wild-type data are from Fig. 4. Data represent means $\pm$ SD of 5 independent experiments. A two-sided Mann-Whitney *U* test was used to calculate *P* values. Source data are provided as a Source Data file.

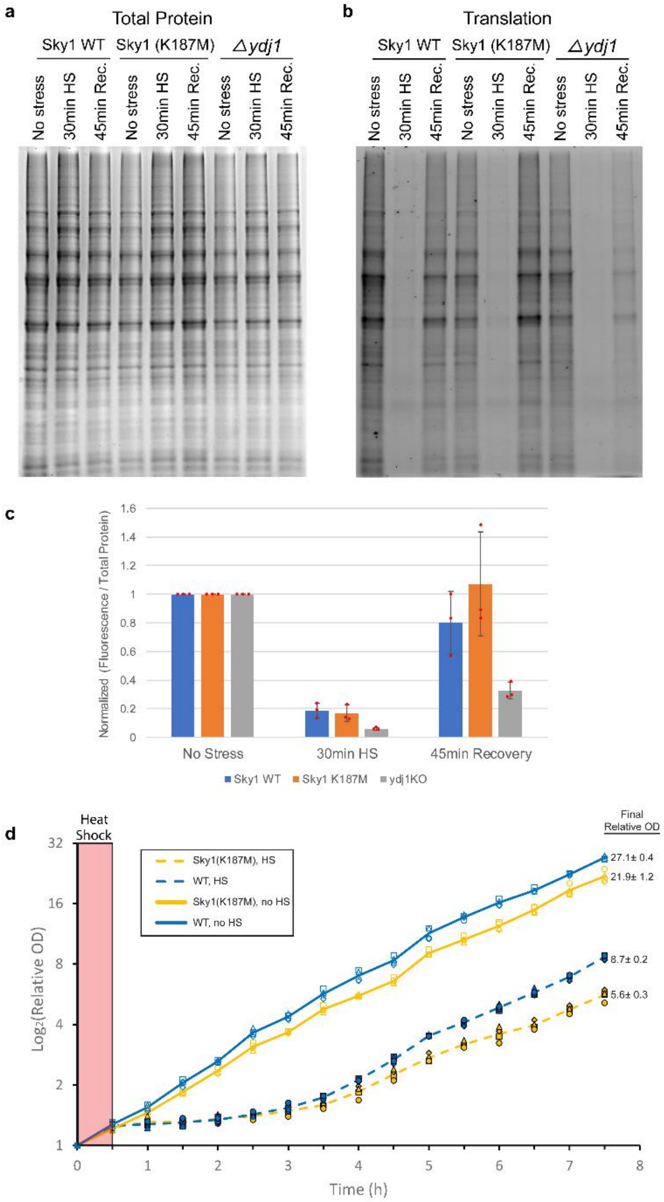

Supplementary Figure 6: Inactivation of Sky1's kinase activity slows recovery of growth after heat stress, but not resumption of translation.
(**a,b**) *sky1*^{K187M} cells show normal recovery of translation after heat stress. Coomassie staining (a) was used to detect total protein, while the methionine analog HPG was used to monitor translation activity (b). A *ydj1*Δ strain, which was previously reported to show delayed resumption of translation, was included as a control. (**c**) Quantification of the ratio of total protein to translational activity in wild-type (blue), *sky1*^{K187M} (orange), or Δ*ydj1* (grey) cells. Data represent means ± SD of 3 independent experiments. (**d**) *sky1*^{K187M} cells show slower growth after heat shock. The optical densities of *SKY1* (WT; blue) and *sky1*^{K187M} (yellow) strains were monitored with (dashed lines) and without (solid lines) 30 minutes of heat shock. Symbols (square, triangle, diamond, and circle) represent the four replicates under each condition. Data represent the means ± SD of four independent cultures. Source data are provided as a Source Data file.

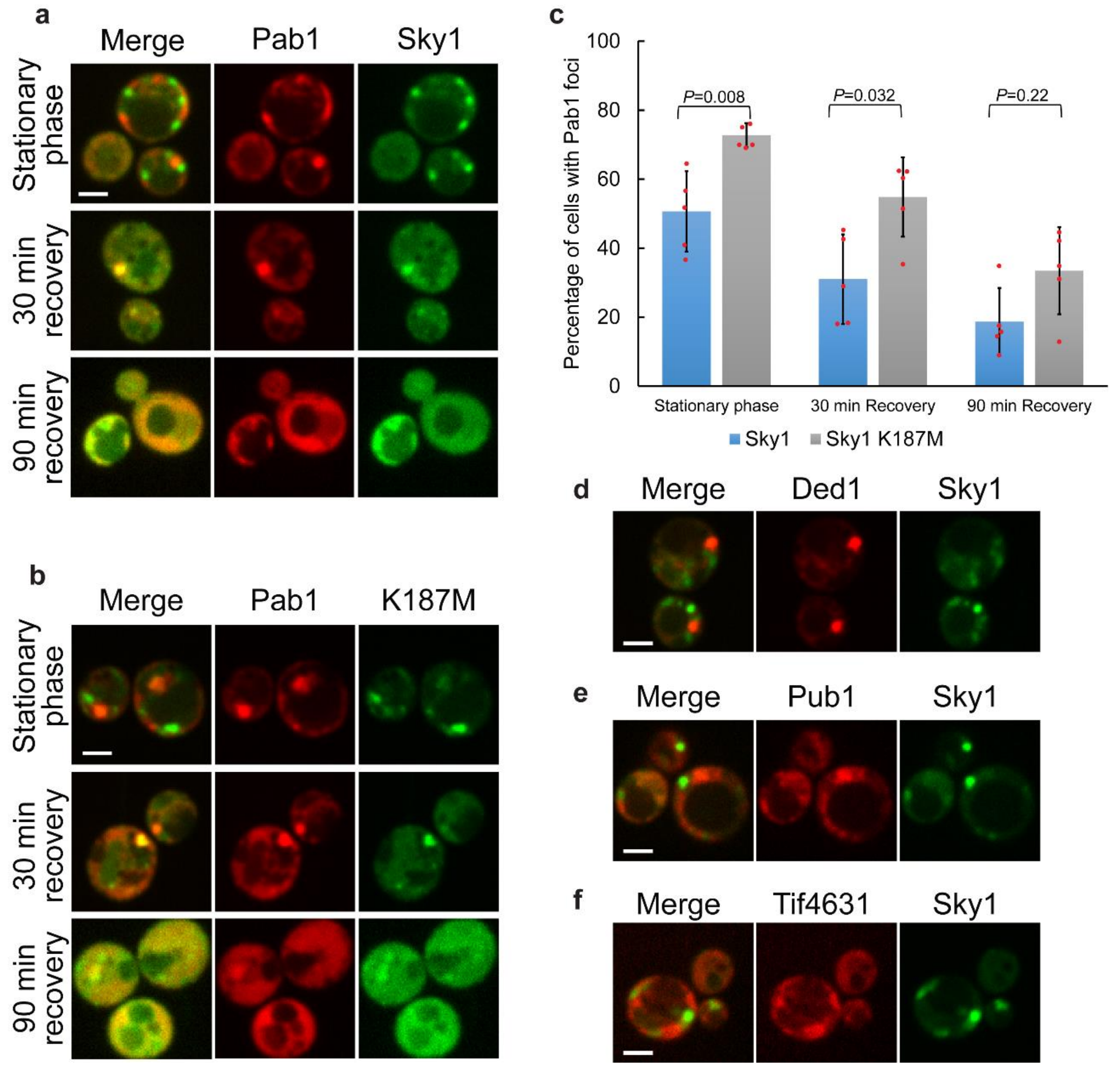

Supplementary Figure 7: Inactivation of Sky1's kinase activity slows stress granule dissolution during recovery from prolonged stationary phase.
(**a,b**) Cell expressing Pab1-mCherry and either Sky1-GFP (a) or Sky1^{K187M}-GFP (b) from the corresponding endogenous loci were grown in liquid culture for 5 days to achieve prolonged stationary phase. Cells were then diluted into fresh medium to allow for recovery. Cells were visualized by fluorescence microscopy before and during recovery. Scale bar, 2 µm. (**c**) Quantification of the percentage of cells with Pab1 foci after prolonged stationary phase and during recovery. Data represent means $\pm$ SD of 5 independent experiments. A two-sided Mann-Whitney *U* test was used to calculate *P* values. (**d-f**) Sky1 shows limited colocalization with other stress granule markers during prolonged stationary phase. Cells expressing Ded1-mCherry (d), Pub1-mCherry (e), or Tif4631-mCherry (f) were grown in liquid culture for 5 days to achieve prolonged stationary phase, and then visualized by fluorescence microscopy. Scale bar, 2 µm. Source data are provided as a Source Data file.

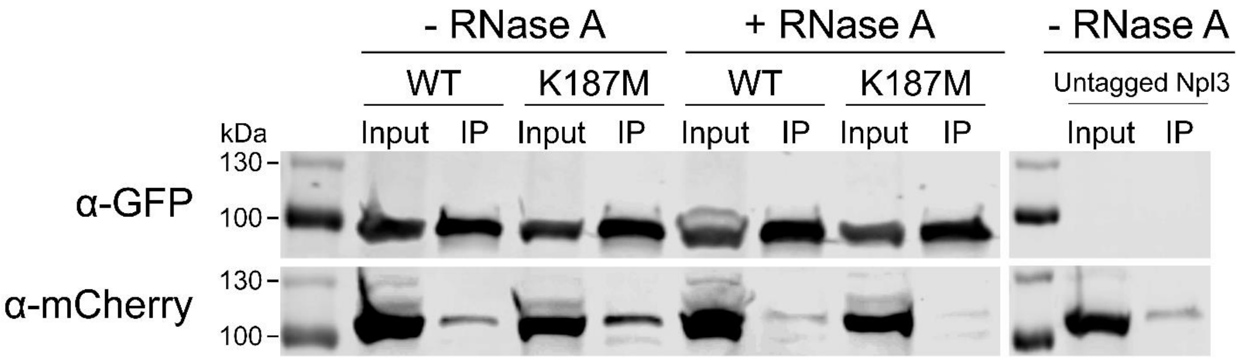

Supplementary Figure 8: Co-immunoprecipitation of Pab1 with Npl3 is enhanced upon Sky1 inactivation.

Lysates from cells expressing Npl3-GFP, Pab1-mCh and either wild-type Sky1 or Sky1^{K187M} were subjected to immunoprecipitation with anti-GFP magnetic beads, with or without treatment with RNase A. Immunoprecipitations were eluted in one-fifth the volume of the initial input to facilitate visualization. Total cells lysates (Input) and immunoprecipitated samples (IP) were analyzed by western blot with anti-GFP or anti-mCherry antibodies. As a negative control to ensure that the immunoprecipitation of Pab1 was not a result of non-specific binding to the beads, cells expressing untagged Npl3 were subjected to immunoprecipitation; this control revealed a low level of non-specific pull-down of Pab1. Source data are provided as a Source Data file.

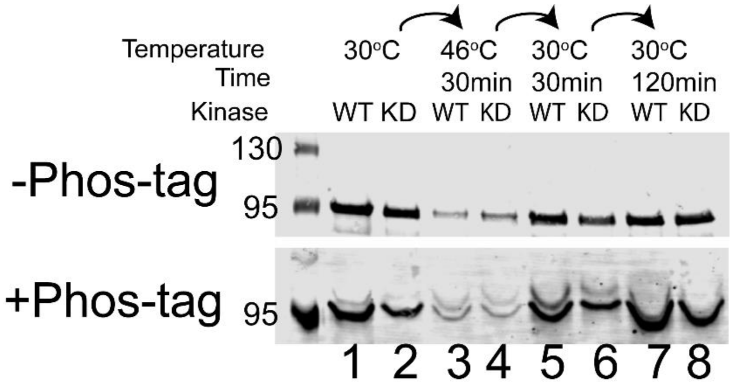

Supplementary Figure 9: Npl3 stress-specific occurs at S411

Yeast expressing Npl3(S411A)-mCherry-Hisx8 and either wild-type Sky1 (WT, odd lanes) or Sky1^{K187M} (KD, even lanes) from the corresponding endogenous loci were grown to mid-log phase in YPD, and subjected to a heat shock and recovery. Cells were analyzed by western blotting with and without the addition of Phos-tag. Source data are provided as a Source Data file.

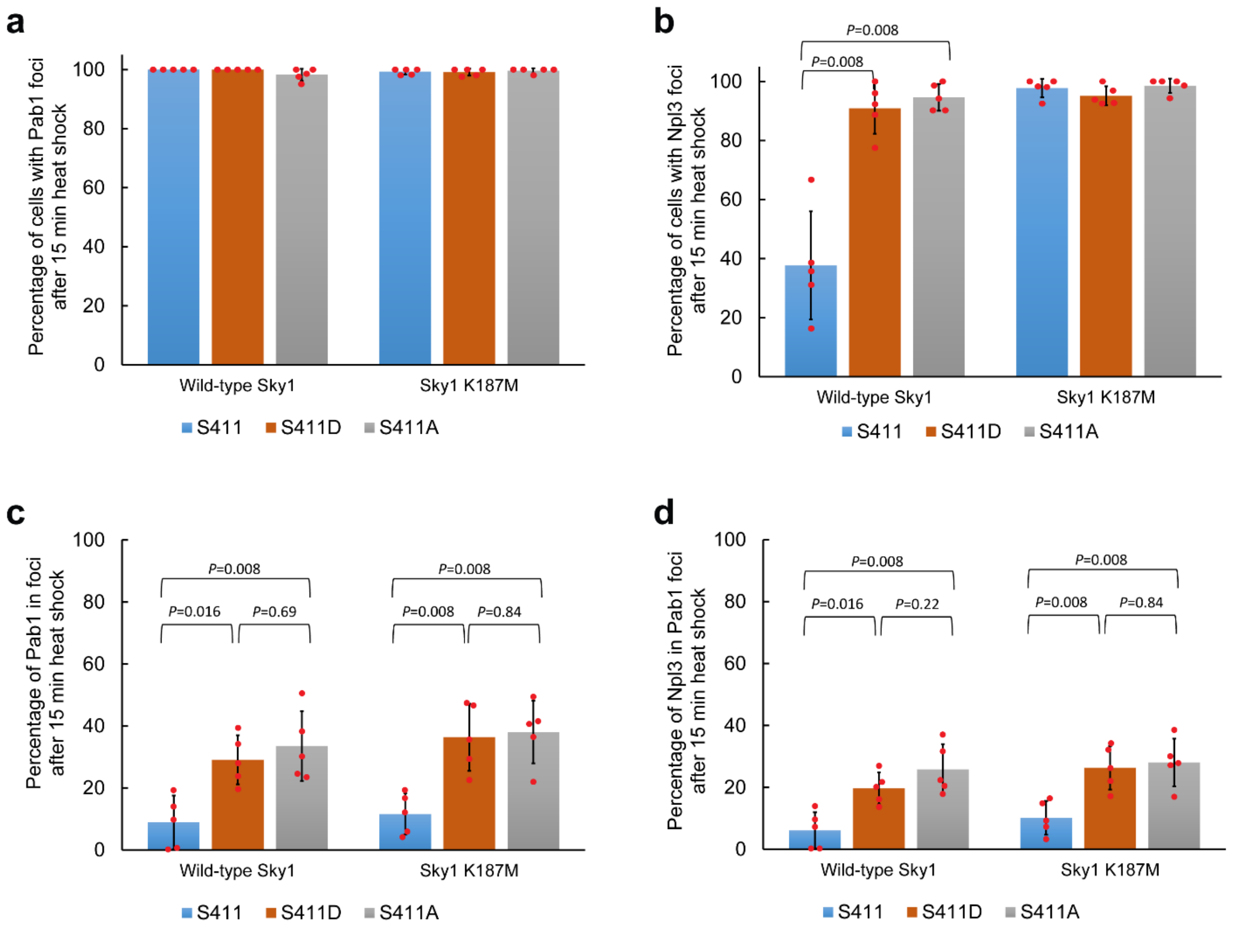

Supplementary Figure 10: Npl3 phosphomimetic and non-phosphorylatable mutations accelerate Pab1 and Npl3 recruitment into granules.

(**a,b**) Quantification of the percentage of cells with Pab1 (a) or Npl3 (b) foci after 15 min heat shock for wild-type *SKY1* or *sky1*^{K187M} cell expressing Pab1-mCherry and GFP-tagged versions of Npl3 (blue), Npl3^{S411D} (orange), or Npl3^{S411A} (grey). Data represent means $\pm$ SD of 5 independent experiments. A two-sided Mann-Whitney *U* test was used to calculate *P* values. (**c**) Percentage of Pab1 protein in foci after 15 min heat shock. Data represent means $\pm$ SD of 5 independent experiments. A two-sided Mann-Whitney *U* test was used to calculate *P* values. (**d**) Percentage of Npl3 protein colocalizing with Pab1 foci after 15 min heat shock. Data represent means $\pm$ SD of 5 independent experiments. A two-sided Mann-Whitney *U* test was used to calculate *P* values. Source data are provided as a Source Data file.

Supplementary Table S1: Yeast strains

Strain Name	Genotype
yER1171	MAT**a** his3D1 leu2D0 met15D0 ura3D0 SKY1-GFP::HIS3 PAB1-mCherry::URA3
yER1404	MAT**a** his3D1 leu2D0 met15D0 ura3D0 PAB1-mCherry::URA3
yER1001	MAT**a** his3D1 leu2D0 met15D0 ura3D0 SKY1-GFP::HIS3
yER1752	MAT**a** his3D1 leu2D0 met15D0 ura3D0 SKY1-dPrLD(aa388-457)-GFP::HIS3
yER2166	MAT**a** his3D1 leu2D0 met15D0 ura3D0 SKY1(N-term PrLD)-GFP::HIS3 (PrLD aa388-457 inserted after G2, and deletion of PrLD in original location)
yER2174	MAT**a** his3D1 leu2D0 met15D0 ura3D0 SKY1(C-term PrLD)-GFP::HIS3 (deletion of PrLD in original location aa388-457, PrLD inserted after H742)
yER2144	MAT**a** his3D1 leu2D0 met15D0 ura3D0 SKY1(Cdc39 PrLD)-GFP::HIS3 (Sky1 PrLD (aa388-457) replaced with Cdc39 PrLD (aa966-1092))
yER2227	MAT**a** his3D1 leu2D0 met15D0 ura3D0 SKY1(Ded1 PrLD)-GFP::HIS3 (Sky1 PrLD (aa388-457) replaced with Cdc39 PrLD (aa1-97))
yER2204	MAT**a** his3D1 leu2D0 met15D0 ura3D0 SKY1(cPFD#1)-GFP::HIS3<br>(Sky1 PrLD (aa388-457) replaced with cPFD#1)
yER2205	MAT**a** his3D1 leu2D0 met15D0 ura3D0 SKY1(cPFD#2)-GFP::HIS3<br>(Sky1 PrLD (aa388-457) replaced with cPFD#2)
yER2206	MAT**a** his3D1 leu2D0 met15D0 ura3D0 SKY1(cPFD#3)-GFP::HIS3<br>(Sky1 PrLD (aa388-457) replaced with cPFD#3)
yER2095	MAT**a** his3D1 leu2D0 met15D0 ura3D0 SKY1-GFP::KanMx PAB1-mCherry::URA3
yER1551	MAT**a** his3D1 leu2D0 met15D0 ura3D0 SKY1(K187M)-GFP::HIS3 PAB1-mCherry::URA3
yER2118	MAT**a** his3D1 leu2D0 met15D0 ura3D0 NPL3-GFP::HIS3 PAB::PAB1-mCherry::URA3
yER2160	MAT**a** his3D1 leu2D0 met15D0 ura3D0 SKY1(K187M) NPL3-GFP::HIS3 PAB1-mCherry::URA3

yER1746	MAT**a** his3D1 leu2D0 met15D0 ura3D0 SKY1-GFP::HIS3 NPL3-mCherry-Hisx8::KanMX
yER1747	MAT**a** his3D1 leu2D0 met15D0 ura3D0 SKY1(K187M)-GFP::HIS3 NPL3-mCherry-Hisx8::KanMX
yER1889	MATa his3D1 leu2D0 met15D0 ura3D0 NPL3(S411D)-GFP::HIS3 PAB1-mCherry::URA3
yER1894	MATa his3D1 leu2D0 met15D0 ura3D0 NPL3(S411A)-GFP::HIS3 PAB1-mCherry::URA3
yER2085	MATa his3D1 leu2D0 met15D0 ura3D0 SKY1(K187M) NPL3(S411D)-GFP::HIS3 PAB1-mCherry::URA3
yER2086	MATa his3D1 leu2D0 met15D0 ura3D0 SKY1(K187M) NPL3(S411A)-GFP::HIS3 PAB1-mCherry::URA3
yER2145	MAT**a** his3D1 leu2D0 met15D0 ura3D0 PAB1-mCherry::URA3 hsp104::HIS3
yER1541	MAT**a** his3D1 leu2D0 met15D0 ura3D0 SKY1-GFP::HIS3 PUB1::PUB1-mCherry::URA3
yER1507	MAT**a** his3D1 leu2D0 met15D0 ura3D0 SKY1-GFP::HIS3 TIF4631-mCherry::URA3
yER1799	MAT**a** his3D1 leu2D0 met15D0 ura3D0 SKY1-GFP::HIS3 DED1-mCherry::URA3
yER1461	MAT**a** his3D1 leu2D0 met15D0 ura3D0 SKY1-GFP::HIS3 NPL3-mCherry-URA
yER1553	MAT**a** his3D1 leu2D0 met15D0 ura3D0 SKY1(K187M)-GFP::HIS3 NPL3-mcherry::URA3
yER2147	MAT**a** his3D1 leu2D0 met15D0 ura3D0 SKY1(dPrLD (aa388-457))-GFP::HIS3 NPL3-mCherry::URA3
yER2203	MAT**a** his3D1 leu2D0 met15D0 ura3D0 SKY1(N-Term PrLD)-GFP::HIS3 NPL3-mCherry::URA3
yER2207	MAT**a** his3D1 leu2D0 met15D0 ura3D0 SKY1(C-term PrLD)-GFP::HIS3 NPL3-mCherry::URA3
yER2211	MAT**a** his3D1 leu2D0 met15D0 ura3D0 SKY1(N-Term PrLD)-GFP::HIS3 PAB1-mCherry::URA3
yER2202	MAT**a** his3D1 leu2D0 met15D0 ura3D0 SKY1(C-term PrLD)-GFP::HIS3 PAB1-mCherry::URA3

yER2096	MAT**a** his3D1 leu2D0 met15D0 ura3D0 SKY1::GFP::KanMx DED1-mcherry-Ura3
yER2124	MAT**a** his3D1 leu2D0 met15D0 ura3D0 SKY1::GFP::KanMx PUB1-mcherry-Ura3
yER1550	MAT**a** his3D1 leu2D0 met15D0 ura3D0 SKY1(K187M)-GFP::HIS3 PUB1-mcherry::URA3
yER1801	MAT**a** his3D1 leu2D0 met15D0 ura3D0 SKY1(K187M)-GFP::HIS3 DED1-mcherry::URA3
yER2118	MATa his3D1 leu2D0 met15D0 ura3D0 NPL3-GFP::His3 PAB1-mCherry::URA3
yER2160	MATa his3D1 leu2D0 met15D0 ura3D0 SKY1(K187M) NPL3-GFP::His3 PAB1-mCherry::URA3
yER2161	MATa his3D1 leu2D0 met15D0 ura3D0 NPL3(S411A)-mCherry-Hisx8::KanMx
yER2162	MATa his3D1 leu2D0 met15D0 ura3D0 SKY1(K187M) NPL3(S411A)-mCherry-Hisx8::KanMx
yER2260	MAT**a** his3D1 leu2D0 met15D0 ura3D0 SKY1(dPrLD)-GFP::HIS3 PAB1-mCherry::URA3
yER2261	MAT**a** his3D1 leu2D0 met15D0 ura3D0 SKY1(cPFD#1)-GFP::HIS3 PAB1-mCherry::URA3
yER2262	MAT**a** his3D1 leu2D0 met15D0 ura3D0 SKY1(cPFD#2)-GFP::HIS3 PAB1-mCherry::URA3
yER2263	MAT**a** his3D1 leu2D0 met15D0 ura3D0 SKY1(cPFD#3)-GFP::HIS3 PAB1-mCherry::URA3
yER2264	MAT**a** his3D1 leu2D0 met15D0 ura3D0 SKY1(Ded1 PrLD)-GFP::HIS3 PAB1-mCherry::URA3
yER2265	MAT**a** his3D1 leu2D0 met15D0 ura3D0 SKY1(Cdc39 PrLD)-GFP::HIS3 PAB1-mCherry::URA3
yER2266	MAT**a** his3D1 leu2D0 met15D0 ura3D0 arg1::HIS3 SKY1::Sky1(K187M)

Supplementary Table S2: Plasmids

Plasmid Name	Description
pER843	*LEU2*,cen, P_{SUP35}-GFP
pER1588	*LEU2*,cen, P_{SUP35}-GFP-Sky1 PrLD
pER1924	*LEU2*,cen, P_{GPD}-Sky1-GFP *K740Q mutation from GFP collection
pER1943	*LEU2*,cen, P_{GPD}-Sky1(K187M)-GFP *K740Q mutation from GFP collection
pER2050	*LEU2*,cen, P_{GPD}-GFP
pER1965	*LEU2*,cen, P_{SUP35}-GFP-cPFD#1
pER1947	*LEU2*,cen, P_{SUP35}-GFP-cPFD#2
pER1948	*LEU2*,cen, P_{SUP35}-GFP-cPFD#3

Supplementary Table S3: Oligonucleotides used for strain construction

Oligo number	Used in yERxxx construction	Oligo DNA Sequence
3018	1171,1405,1551, 2118, 2160, 2211, 2202,2095,1889, 1894,2085,2086, 2145,2260,2261, 2262,2263,2264, 2265	CTGCCTATGAGTCTTTCAAAAAGGAGCAAGAACAACAAACTGAGCAAGCTatggtgagcaagggcgagg
3036	1171,1405,1551, 2118, 2160, 2211, 2202,2095,1889, 1894,2085,2086, 2145, 2260,2261, 2262,2263,2264, 2265	AAAAAGATGATAAGTTTGTTGAGTAGGGAAGTAGGTGATTACATAGAGCAgtatcacgaggccctttcg
3605	1541,1550,2124	GAATGACCAACAACAACCGGTTATGTCTGAGCAACAACAGCAACAGCAGCAACAGCAGCAACAACAAatggtgagcaagggcgagg
3606	1541,1550,2124	GCCTCTCTTTATTCTTTCTTTTTGTTTCATTCCACTTTTCTTCATAATATgtatcacgaggccctttcg
3603	1507,1552	CCGCAACAAATATGTTCAGTGCATTAATGGGAGAAAGTGATGACGAAGAGatggtgagcaagggcgagg
3604	1507,1552	CATCCTTGTATCCAAGTGACATTTTCGATACTTAACATGATCTATTCATGgtatcacgaggccctttcg
3975	1799,1801,2096	AGTCTTCTGGCTGGGGTAACAGCGGTGGTTCAAACAACTCTTCTTGGTGGatggtgagcaagggcgagg
3976	1799,1801,2096	AGACATGCTAGAGCAGAAAACGAAGAATCCTCACCCTAGTTTGTCTGAAAgtatcacgaggccctttcg
3566	1551,1550,1552, 1801	taagaaagctgggatggggccacttctccaccgtttggttggcaaaggatATGGTAAACAACACTCACGTTGCTATGATGATTGTTCGGGGCGACAAAGT
3567	1551,1550,1552, 1801	ccctcaacatctccaatctccatcaacacgttttctggtttaatatctgtGTGTATAATACCACACCGCCTATG

4483	2095,2096,2124	ACCCCCTTTTGAGGTTGAAGAGATAGAGTAAAGAAGAAGTGTAGACATTAatgAGTAAAGGAGAAGAACTTTTCACTGGAG
4484	2095,2096,2124	AAACAGAAAAAAAAGTAAAAGGCAAGGCAAAATAAAGGTATAAAGGTAACAGTATAGCGACCAGCATTCAC
3560	1461,1553,2147, 2203,2207	GAGATGCATACAGAACCAGAGATGCTCCACGTGAAAGATCACCAACCAGGatggtgagcaagggcgagg
3561	1461,1553,2147, 2203,2207	TCATATCTTTTGTTAATTTCTCCTTTTTTTTTCTCAACTATATAAATGGCgtatcacgaggccctttcg
3800	1746,1746,2161, 2162,2164,2165	TTAAAACAATTCATATCTTTTGTTAATTTCTCCTTTTTTTTTCTCAACTATATAAATGGCttacagtatagcgaccagcattcac
3844	1746,1746,2161, 2162,2164,2165	GAACGCGCCGAGGGCCGCCACTCCACCGGCGGCATGGACGAGCTGTACAAGCATCACCACCATCATCATCACCATTAGGGCGCGCCACTTCTAAATAAG
3153	2118,2160,1746, 1747,1889,1894, 2085,2086	TCATATCTTTTGTTAATTTCTCCTTTTTTTTTCTCAACTATATAAATGGCgaattcgagctcgtttaaactgg
4089	2161,2162,1894, 2086	GAGATGCATACAGAACCAGAGATGCTCCACGTGAAAGAGCTCCAACCAGGcggatccccgggttaattaacag
4090	1889,2085	GAGATGCATACAGAACCAGAGATGCTCCACGTGAAAGAGATCCAACCAGGcggatccccgggttaattaacag
2709	2145	ACAAAGAAAAAAGAAATCAACTACACGTACCATAAAATATACAGAATATcagattgtactgagagtgcacc
2710	2145	TATTATATTACTGATTCTTGTTCGAAAGTTTTTAAAAATCACACTATATTAAActacataagaacacctttggtggag
3810	1752	TGTCAAAAAGATGCTTTAGAAGACCTAGACGTCATACAATTATCACAGGGTTCATAAATGAAGACAGTAATGATAACAACAATAATGATAATAGTAAAAA
3811	1752	TTTTTACTATTATCATTATTGTTGTTATCATTACTGTCTTCATTTATGAACCCTGTGATAATTGTATGACGTCTAGGTCTTCTAAAGCATCTTTTTGACA
4448	2166,2211	TTTTGAGGTTGAAGAGATAGAGTAAAGAAGAAGTGTAGACATTAATGGGTAGTATGCCCTGCGGCTC

4449	2166,2211	TCAGCCAAATGAGCGCTTTTAGTCACAAACCCAGGATAGTTAATTGATGACATTATGTCCTCGTTATTATTGTTGTTACTGTTG
4452	2174,2202	GTTCCGATATCCCCGGATGGTTTGAAGAAGTCCGCGATCATAAAAGACATAGTATGCCCTGCGGCTC
4453	2174,2202	actccagtgaaaagttcttctcctttactgttaattaacccggggatccgCATTATGTCCTCGTTATTATTGTTGTTACTGTTG
4544	2144	TGTCAAAAAGATGCTTTAGAAGACCTAGACGTCATACAATTATCACAGGGactttcgaagttgaggttttattaaaatcttttaatttgaccac
4545	2144	TTTTTACTATTATCATTATTGTTGTTATCATTACTGTCTTCATTTATGAAaaataccctcttcaagtcagggtgg
4567	2204	TGTCAAAAAGATGCTTTAGAAGACCTAGACGTCATACAATTATCACAGGGATGTCTCAAGCAGCATCTACTAAACAATC
4569	2205	TGTCAAAAAGATGCTTTAGAAGACCTAGACGTCATACAATTATCACAGGGATGTCTACTTCTCAAACTATGCCAATG
4570	2206	TGTCAAAAAGATGCTTTAGAAGACCTAGACGTCATACAATTATCACAGGGATGTCTGATCAACATAAAACTAATTCTCAACAAAGAC
4568	2204,2205,2206	TTTTTACTATTATCATTATTGTTGTTATCATTACTGTCTTCATTTATGAAACCTTGAGACTGTGGTTGGAAAC
4563	2227	TGTCAAAAAGATGCTTTAGAAGACCTAGACGTCATACAATTATCACAGGGatggctgaactgagcgaacaag
4564	2227	TTTTTACTATTATCATTATTGTTGTTATCATTACTGTCTTCATTTATGAATGGAGCTGGGACATGTTTGC
291	2266	tgttctaatatacacggatacaaaagaaatacacataattgcataaaataGTCATAACACAGTCCTTTCCCG
292	2266	gaggtggaggaagagatcgttatctatcttgaggcgatgaactagcggacCACCGCATATGATCCGTCG

Supplementary Table S4: Oligonucleotides used in plasmid construction

Oligo number	Used for pERxxx construction	Oligo DNA Sequence
3407	1588	GTCGATGCTAagatctccttaCATTATGTCCTCGTTATTATTGTTGTTACTGTTG
2289	1588	GAGCTACTGGATCCACAatgtctAGTATGCCCTGCGGCTCAAG
4231	1924,1943	ttcgacggattctagATGGGTTCATCAATTAACTATCCTGGG
4309	1924,1943,2050	aattacatgactcgagctatttgtatagttcatccatgccatgtgt
4591	2050	ttcgacggattctagaatgAGTAAAGGAGAAGAACTTTTCACTGG
4305	1965	ttctgctggtGGATCCATGTCTCAAGCAGCATCTACTAAACAATCTACTG
4307	1947	ttctgctggtGGATCCATGTCTACTTCTCAAACTATGCCAATGC
4308	1948	ttctgctggtGGATCCATGTCTGATCAACATAAAACTAATTCTCAACAAAGAC
4306	1965,1947,1948	tgcaagaaatAGATCTtcaACCTTGAGACTGTGGTTGGAAAC
